# Novel biomarkers distinguish heart failure with preserved vs reduced ejection fraction

**DOI:** 10.1093/eschf/xvaf011

**Published:** 2026-01-08

**Authors:** Camilla Hage, Annika Mang, Jean-Claude Daubert, Erwan Donal, Peder L Myhre, Cecilia Linde, Lars H Lund

**Affiliations:** Cardiology Unit, Department of Medicine, Karolinska Institutet, Stockholm, Sweden; Department of Cardiology, Karolinska University Hospital, Stockholm SE-171 64, Sweden; Roche Diagnostics GmbH, Penzberg, Germany; Département de Cardiologie & CIC-IT U 804, Centre Hospitalier Universitaire de Rennes, Rennes, France; Département de Cardiologie & CIC-IT U 804, Centre Hospitalier Universitaire de Rennes, Rennes, France; Department of Cardiology, Division of Medicine, Akershus University Hospital, Lørenskog, Norway; K.G. Jebsen Center for Cardiac Biomarkers, Institute of Clinical Medicine, University of Oslo, Oslo, Norway; Cardiology Unit, Department of Medicine, Karolinska Institutet, Stockholm, Sweden; Department of Cardiology, Karolinska University Hospital, Stockholm SE-171 64, Sweden; Cardiology Unit, Department of Medicine, Karolinska Institutet, Stockholm, Sweden; Department of Cardiology, Karolinska University Hospital, Stockholm SE-171 64, Sweden

**Keywords:** HFrEF, HFpEF, biomarker, pathophysiology, oxidative stress, phenotype

## Abstract

**Introduction:**

Heart failure (HF) with preserved (HFpEF) and reduced (HFrEF) ejection fraction may be driven by different pathophysiologies. We explored novel biomarkers, associations with clinical characteristics, discrimination between LVEF categories and associations with outcomes.

**Methods and results:**

In HFpEF(*n* = 76) and HFrEF(*n* = 36), 19 plasma biomarkers were measured including seven novel research assays for ANGPT2, BMP10, DKK3, FABP3, FGF23, IGFBP7, and MYBPC3. Heart failure with preserved ejection fraction patients were older (73 vs 63 years), more often female (50% vs 14%). All seven novel biomarkers except FABP3 tended to be higher in HFrEF vs HFpEF and associated with worse NYHA class and lower eGFR in both LVEF categories. MYBPC3 and FGF23 (higher in HFrEF) discriminated best between LVEF categories (AUC 85.8 and 80.0 respectively). In HFpEF, higher ANGPT2 was associated with worse right (TAPSE: *β* = −1.03;*P* = .04) and left ventricular function (LV-GLS; *β* = 1.29;*P* = .03) and left atrial strain (LA-GLS: *β* = 5.03;*P* < .001) whereas higher IGFBP7 and MYBPC3 with diastolic dysfunction (*E*/*e*ʹ: *β* = 4.09;*P* = .02 and *β* = 1.36;*P* = .01 respectively). All biomarkers except DKK3 were positively associated with the outcome (HFpEF:all-cause death, HF hospitalization;HFrEF: all-cause death, LVAD or heart transplantation). Specifically ANGPT2 [HR 1.45(95% CI 1.00–2.13)] more strongly in HFpEF and IGFBP7 [2.51(0.95–6.64)] more strongly in HFrEFand MYBPC3 [1.62(0.99–2.64)].

**Conclusion:**

Among seven novel biomarker assays, higher MYBPC3 (reflecting muscle injury and myopathy) and FGF23 (endothelial dysfunction, oxidative stress) distinguished HFrEF from HFpEF. Higher MYBPC3 was most prognostic in HFrEF while higher ANGPT2 and IGFBP7 (endothelial dysfunction and oxidative stress) in HFpEF. These hypothesis-generating findings support primary cardiomyocyte injury as a driver of HFrEF and systemic inflammation and oxidative stress as a driver of HFpEF.

**Clinical Trial Registration:**

ClinicalTrials.gov NCT00774709

## Graphical Abstract

**Graphical Abstract:**
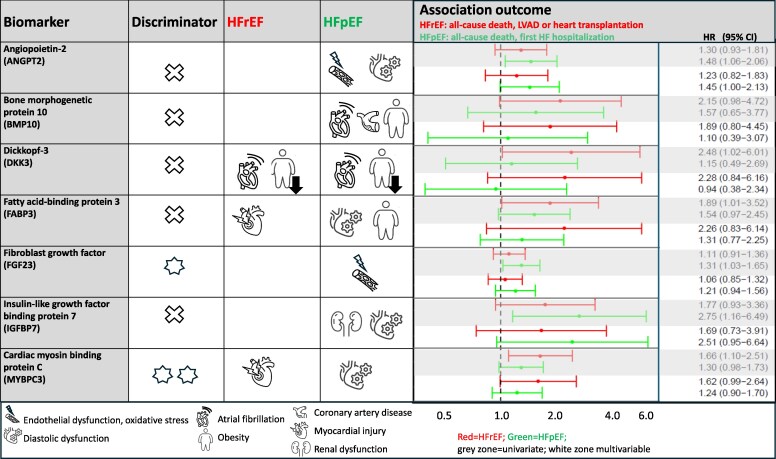

## Introduction

Heart failure (HF) and guideline recommended HF treatment is defined according to the left ventricular ejection fraction (LVEF) as reduced (LVEF ≤40%; HFrEF), mildly reduced (LVEF 41%–49% HFmrEF) or preserved LVEF (≥50%; HFpEF).^1^ While in HFrEF successful advances in HF drug and device therapy have been made by blocking neurohormonal activation^1^ in HFpEF, only SGLT2-inhibitors and the non-steroidal mineralocorticoid receptor antagonist finerenone have been shown to improve outcomes.^2,3^

Heart failure LVEF categories may differ in underlying pathophysiological drivers.^4–6^ The paradigm in HFpEF is that non-cardiac co-morbidities result in systemic and endothelial inflammation and microvascular dysfunction driving cardiomyocyte dysfunction and interstitial fibrosis, whereas the HFrEF syndrome primarily is triggered by direct cardiomyocyte injury (e.g. after myocardial infarction or genetic variants affecting cardiomyocytes) initiating systemic neuroendocrine activation.^7,8^ These differences result in partly separate molecular diseases leading to different patterns of cardiac remodeling.^9^

Pathophysiological mechanisms can be reflected by circulating biomarkers. So far biomarkers mirroring inflammation, oxidative stress, vascular dysfunction, matrix remodelling and myocardial injury have provided limited incremental diagnostic and prognostic information over natriuretic peptides (BNP, N-terminal pro-brain natriuretic peptide; NT-proBNP) which presently is the only biomarker recommended for assessing presence and severity of HF.^1^ Heart failure biomarkers and their expression and associations with different LVEF categories and outcomes may further characterize HFpEF and HFrEF pathophysiology and potential treatment targets.

We assessed seven biomarkers by novel research assays (not previously assessed in relation to HF) in HFpEF and HFrEF: *(i) Angiopoietin-2 (ANGPT2, UniProt ID O15123), (ii) bone morphogenetic protein 10 (BMP10, UniProt ID O95393)*, *(iii) Dickkopf-3 (DKK3, UniProt ID Q9UBP4)*, *(iv) fatty acid-binding protein 3 (FABP3, UniProt ID P05413), (v) Fibroblast growth factor (FGF23, UniProt ID Q9GZV9)*, *(vi) insulin-like growth factor binding protein 7 (IGFBP7, UniProt Q16270) and (vii) cardiac myosin binding protein C (MYBPC3, cMyBP-C, UniProt ID Q14896).*

*We explored* (i) their ability to discriminate HFpEF from HFrEF; (ii) associations with clinical characteristics in HFpEF and HFrEF; (iii) associations with echocardiographic indices in HFpEF patients; and (iv) association with clinical outcomes.

## Methods

### Patients

Patients with HFpEF were enrolled from the Karolinska Rennes (KaRen) prospective observational multicentre study characterizing patients with HFpEF, previously described.^10^ In brief, 539 patients were enrolled, whereof 86 in the biomarker sub-study, between 21 of May 2007 and 29 of December 2011. Patients presented to hospital with acute signs and symptoms of HF according to the Framingham criteria, NT-proBNP >300 ng/l and LVEF ≥45% assessed within 72 h from presentation. Blood sampling and echocardiography were performed in stable condition 4–8 weeks after hospital presentation and followed until 30 September 2012 when vital status was assessed by telephone contact or by the Swedish National Patient Register and Population Register. The primary composite outcome was defined as time to death from any cause or first hospitalization due to HF. All HF hospitalizations were defined according to clinical judgment by the local investigator and additionally centrally adjudicated.

Patients with HFrEF (*n* = 79) were enrolled from the MetAnEnd study recruiting patients with LVEF <40%, referred to the Karolinska University Hospital between January 2009 and September 2014.^11^ The primary composite outcome was all-cause death, LVAD implantation or HTx. Vital status was assessed by the Swedish National Patient and Population Registers and implantation of LVAD or HTx by patient charts in December 2014. This study did not comprise full Doppler echocardiographic assessment.

Left ventricular hypertrophy (LVH) was defined as left ventricular mass index (LVMI) ≥ 95 g/m^2^ in women and ≥115 g/m^2^ in men, respectively.^1^ Left ventricular global longitudinal strain (LV-GLS) is expressed as a negative value, the more negative values indicate better cardiac function. Estimated glomerular filtration rate (eGFR) was calculated according to the CKD-EPI 2021 equation.

### Sample preparation and laboratory analyses

Venous plasma samples were collected in ethylenediaminetetraacetic acid tubes, centrifuged and plasma was stored in aliquots in −70°C until analysis.

### Seven novel biomarker assays

Circulating protein levels of ANGPT2, BMP10, DKK3, FABP3, FGF23, IGFBP7, and MYBPC3 were analysed using non-commercial research use only (RUO) assays on a cobase 601 instrument. All employ a quantitative sandwich format, employing one biotinylated monoclonal antibody for capturing and a second ruthenylated monoclonal antibody for detection. Remaining biomarker assays are presented in Supplementary Table S1.

### Statistics

Descriptive data are expressed as median and 25th and 75th quantile or absolute numbers and percentages (%) as appropriate. Statistical comparison for categorical variables was performed by Fishers exact test, and for continuous variables by Wilcoxon rank-sum test. Novel and traditional (NT-proBNP, eGFR) biomarkers were not normally distributed as identified visually with a histogram and therefore log-transformed prior to analysis.

Univariate and bivariate (exploring combinations of two biomarkers) analyses were performed to discriminate between HFpEF and HFrEF. For univariate analysis, the log-transformed concentrations of the biomarkers were used to discriminate HFpEF from HFrEF. For bivariate analyses log-transformed biomarkers were combined using a logistic regression model. Associations between clinical characteristics and the seven novel biomarkers were assessed by mean comparison within HFrEF and HFpEF groups and separately by applying Wilcoxon tests. In HFpEF, association between echocardiographic variables and biomarkers was performed in multivariable linear regression analyses, adjusted for age, sex and atrial fibrillation/flutter (AFF). Linear regression analyses modelled by HF class were performed to explore association between each of the seven novel biomarkers and eGFR and NT-proBNP, respectively, and the impact of LVEF category. Association between the seven novel biomarkers and New York Heart Association (NYHA) class are presented in box plots and compared by Wilcoxon rank-sum test.

Follow-up time is presented as median and first and third quartiles. Associations between log-transformed biomarkers and outcome were determined with Kaplan-Meier and Cox proportional hazards model, the latter presented as hazard ratio (HR) and 95% confidence interval (CI). The multivariable Cox regression model included age, sex and eGFR eGFR ≥/<60 ml/min/1.73 m^2^ considered as clinically significant covariates. *P*-values were 2-sided and statistical significance was set at <.05. All analyses were performed using R version 3.6.3.

## Results

### Clinical characteristics in Hear failure with preserved and Hear failure with reduced ejection fraction (Table 1)

Clinical characteristics of the 76 HFpEF and 36 HFrEF patients with available blood samples are presented in *Table 1*. heart failure with preserved ejection fraction patients were significantly older (73 vs 63 years), more often female (50% vs 14%) with higher systolic blood pressure (145 vs 115 mmHg; all *P* < .001). Co-morbidities differed nominally between LVEF categories with a prevalence of AFF in 61% vs 47% and diabetes in 34% vs 39% in HFpEF compared with HFrEF.

**Table 1:** Baseline clinical characteristics in patients with heart failure with preserved and heart failure with reduced ejection fraction

Variable	HFpEF (*n* = 74)	HFrEF (*n* = 36)	*P*-value
*Demographics*			
Age (years)	73 (66.3;79.0)	63 (49.8;65.3)	<.001
Female sex	37 (50%)	5 (14%)	<.001
*Physical exam*			
BMI (kg/m^2^)	27.9 (25;33.1)	27.5 (23.8;30.7)	.279
SBP (mm Hg)	145 (125;150)	115 (105;127)	<.001
HR (beats/min)	70 (60.3;81.8)	70 (60;71)	.147
NYHA class			<.001
I	17 (23%)	1 (3%)	
II	39 (53%)	2 (6%)	
III	18 (24%)	30 (83%)	
IV	0 (0%)	3 (8%)	
*Medical history*			
CAD documented in HFpEF/ischaemic aetiology in HFrEF	23 (31%)	16 (44%)	.204
Atrial fibrillation/flutter	45 (61%)	17 (47%)	.220
Diabetes mellitus	25 (34%)	14 (39%)	.673
COPD	13 (18%)	3 (8%)	.257
Cancer	13 (18%)	1 (3%)	.033
CABG	9 (12%)	9 (25%)	.104
PCI	8 (11%)	9 (25%)	.089
*Medical treatment*			
ARB	24 (32%)	12 (33%)	1.000
ACEi	38 (51%)	24 (67%)	.154
MRA	17 (23%)	25 (69%)	<.001
Loop diuretic	54 (73%)	34 (94%)	.010
Beta blocker	59 (80%)	36 (100%)	.002
Calcium channel blocker	23 (31%)	2 (6%)	.003
Implanted cardiac defibrillator	—	32 (100%)	1.000
Statin	32 (43%)	14 (39%)	.686
*Echocardiography*			
LVEF	63 (58;68)	21.3 (15;25.6)	<.001
LV-GLS	−19.5 (−22.3;−15.8)		
LVEDD mm	48.5 (44;53)	65 (60.8;75.8)	<.001
LV end-diastolic diameter	48.5 (43.3;53)		
LV mass indexed	115 (97;144)		
LA volume indexed	43.7 (38.1;55.4)		
LA-GLS	11.4 (4.4;21.3)		
E′ mean velocity	8 (7;10)		
*E*/*E*′ ratio	10.1 (8.2;14)		
*E*/*A* ratio	1.3 (0.9;2.1)		
Isovolumic relaxation time	97.5 (77.8;113)		
E-wave deceleration time	202 (154;229)		
Mitral inflow velocity time integral	23 (16;30)		

ACEi, angiotensin-converting enzyme inhibitor; ARB, angiotensin receptor blocker; BMI, body mass index; CAD, coronary artery disease; CABG, coronary artery bypass graft surgery; COPD, chronic obstructive pulmonary disease; HR, heart rate; GLS, global longitudinal strain; LA, left atrium; LV, left ventricle; LVEF, left ventricular ejection fraction; LVEDD, LV end-diastolic diameter; MRA, mineralocorticoid receptor antagonist; NYHA, New York Heart Association; SBP, systolic blood pressure; PCI, percutaneous coronary intervention.

### Biomarker concentrations

Six out of seven novel biomarkers (ANGPT2, BMP10, FGF23, IGFBP7, and MYBPC3) were significantly or nominally (DKK3) lower in HFpEF vs HFrEF while FABP3 did not differ between LVEF categories (*Table 2*). Additionally, previously studied biomarker concentrations reflecting HF severity, renal function, inflammation and metabolic status were in general lower in HFpEF compared with HFrEF, except for IGF-1 (173 vs 123 µg/l *P* < .001) (*Table 2*). NT-proBNP was 1012 pg/ml in HFpEF and 2596 pg/ml in HFrEF.

**Table 2:** Circulating biomarkers in patients with heart failure with preserved and heart failure with reduced ejection fraction

Biomarker	HFpEF (*N* = 74)	HFrEF (*N* = 36)	*P*-value
ANGPT2 (ng/ml)	3.5 (2.3;6.2)	7.7 (4.1;13.6)	<.001
BMP10 (ng/ml)	2.5 (2;2.9)	3 (2.5;3.8)	<.001
DKK3 (ng/ml)	60.8 (50.9;67.9)	66.9 (54.4;81.8)	.055
FABP3 (ng/ml)	35.9 (29.7;50.4)	39.9 (31.6;54.4)	.24
FGF23 (pg/ml)	263.6 (162.9;431.6)	897.5 (405.2;2447.7)	<.001
IGFBP7 (ng/ml)	130.7 (112.1;154.1)	169.9 (125.8;236.3)	.001
MYBPC3 (pg/ml)	29.7 (17.7;47.1)	80.3 (63.8;158.8)	<.001
NT-proBNP (pg/ml)	1011.6 (464.6;2310.3)	2596.3 (1399.5;4784.6)	<.001
sST2 (µg/l)	23 (16.6;29.9)	37.8 (23.7;52)	<.001
GDF15 (pg/ml)	2087.3 (1458.9;3479.8)	3140.3 (2266.3;5508.5)	.008
Galectin 3 (µg/l)	17.1 (13;21.1)	18.1 (13.3;21.6)	.667
Copeptin (pmol/l)	13.8 (9;22)	27.6 (18.5;44)	<.001
MR-proANP (pmol/l)	310.2 (190;374.8)	390.3 (323.9;557.7)	<.001
MR-proADM (nmol/l)	1.2 (0.9;1.6)	1.3 (1;1.9)	.279
hsTnT (pg/ml)	18.2 (13.2;25.4)	35.3 (24.9;51.6)	<.001
IGF1 (µg/l)	173 (137;197)	123 (95.3;174.3)	<.001
IGF1 SD score	1.2 (0.6;1.8)	−0.6 (−1.6;0.3)	<.001
IGFBP1 (µg/l)	48 (28.3;78)	72.5 (28;101)	.164
Insulin (µU/ml)	11.3 (8.4;17)	12 (7.3;24.5)	.774
Adiponectin (mg/l)	11.8 (8.2;20.1)	10.1 (6.1;20.6)	.831
Leptin (ng/l)	24.2 (10.2;51)	16.6 (8.6;29.1)	.138
HOMA-IR ratio	3.5 (2;5.8)	3.3 (1.5;6.3)	.937
Glucose (mmol/l)	5.6 (5.1;7.4)	5.9 (4.9;7)	.352
Haemoglobin (g/dl)	129.5 (121;139)	137 (124.5;145.3)	.153
Creatinine (µmol/l)	83.5 (72;106.8)	113 (90.5;145.3)	<.001
eGFR (ml/min/1.73 m^2^) (CKD-EPI)	73.7 (54.1;86.2)	62.3 (43.9;76.3)	.071
Potassium (mmol/l)	4 (3.7;4.2)	4.2 (3.9;4.4)	.010
Sodium (mmol/l)	141 (139.3;143)	138 (135.8;139.3)	<.001

ANGPT2, angiopoietin-2; BMP10, bone morphogenetic protein 10; DKK3, Dickkopf-3; eGFR, estimated glomerular filtration rate; FABP3, fatty acid-binding protein 3; FGF23, fibroblast growth factor; GDF15, growth differentiation factor 15; HOMA-IR, homeostatic model assessment-insulin resistance; IGF1, Insulin-like growth Factor 1; IGFBP1, insulin-like growth factor 1 binding protein 1; insulin-like growth factor binding protein 7, IGFBP7; MYBPC3, cardiac myosin binding protein C; MR-proANP, MR-pro-atrial natriuretic peptide; MR-proADM, MR-pro-adrenomedullin; NT-proBNP, N-terminal pro-brain natriuretic peptide; SD, Standard deviation; sST2, soluble suppression of tumorigenicity 2; hsTnT, high sensitive troponin T.

### Biomarkers discriminating heart failure with preserved from Hear failure with reduced ejection fraction (Tables 3 and 4)

Biomarkers in order from highest to lowest discriminating power for HFpEF vs HFrEF are presented in *Table 3* (univariate) and 3b (bivariate). MYBPC3 (AUC 85.8) followed by FGF23 (AUC 80.0) (*Table 3*) were the most prominent (higher in HFrEF). The biomarker combination with the highest discriminative power was MYBPC3 and IGF-1 expressed as SDscore (AUC 90.4; *Table 4*).

**Table 3:** Biomarkers (log-transformed) distinguishing heart failure with preserved from heart failure with reduced ejection fraction

Biomarkers	AUC (95% cI)
MYBPC3	85.8 (78.9–92.6)
FGF23	80.0 (70.7–89.3)
Sodium	78.1 (68.7–87.5)
NT-proBNP	77.9 (68.7–87.0)
Copeptin	75.9 (66.6–85.2)
hsTnT	75.3 (65.3–85.3)
Creatinine	75.0 (66.0–83.9)
sST2	74.2 (63.9–84.6)
MR-proANP	73.3 (62.9–83.7)
ANGPT2	72.6 (62.1–83.1)
BMP10	72.3 (62.0–82.5)
IGF1 SD score	69.1 (53.0–85.2)
IGFBP7	68.8 (57.6–80.0)
GDF15	65.8 (54.4–77.2)
Potassium	65.2 (54.2–76.2)
DKK3	61.3 (49.8–72.9)
Leptin	58.8 (47.6–69.9)
Hemoglobin	58.4 (46.4–70.5)
IGFBP1	58.2 (45.7–70.8)
FABP3	56.9 (45.3–68.6)
MR-proADM	56.4 (44.3–68.5)
Galectin 3	52.6 (41.0–64.1)
Insulin	51.7 (39.2–64.3)
Adiponectin	51.3 (39.3–63.3)
Glucose	44.5 (32.4–56.6)

Ordered by decreasing AUC, based on patients with all biomarkers available.

ANGPT2, Angiopoietin-2; BMP10, none morphogenetic protein 10; CI, confidence interval; DKK3, Dickkopf-3; eGFR, estimated glomerular filtration rate; FABP3, fatty acid-binding protein 3; FGF23, fibroblast growth factor; GDF15, growth differentiation factor 15; IGF1 SD score, insulin-like growth Factor 1 standard deviation score; IGFBP1, insulin-like growth Factor 1 binding protein 1; insulin-like growth factor binding protein 7, IGFBP7; MYBPC3, cardiac myosin binding protein C; MR-proANP, MR-pro-atrial natriuretic peptide; MR-proADM, MR-pro-adrenomedullin; NT-proBNP, N-terminal pro-brain natriuretic peptide; sST2, soluble suppression of tumorigenicity 2; hsTnT, high sensitive troponin T.

**Table 4:** Bivariate combinations of biomarkers (log-transformed) distinguishing heart failure with preserved from heart failure with reduced ejection fraction

Combination of biomarkers	AUC (95% cI)
MYBPC3 + IGF1 SDscore	90.4 (81.8–99.0)
MYBPC3 + Sodium	89.8 (84.2–95.4)
FABP3 + MYBPC3	89.1 (82.7–95.6)
FGF23 + MYBPC3	88.1 (81.7–94.4)
ST2 + MYBPC3	87.8 (81.5–94.1)
Leptin + MYBPC3	87.7 (81.0–94.3)
Galectin 3 + MYBPC3	87.5 (81.0–94.1)
MYBPC3 + NT-proBNP	87.4 (80.9–93.9)
Hemoglobin + MYBPC3	87.3 (81.0–93.6)
IGF1 SD score + Sodium	87.2 (76.9–97.5)
MYBPC3 + MR-proADM	86.7 (80.1–93.3)
ANGPT2 + MYBPC3	86.7 (79.6–93.8)
BMP10 + MYBPC3	86.6 (79.9–93.3)
Copeptin + MYBPC3	86.5 (79.6–93.4)
Insulin + MYBPC3	86.4 (79.8–93.1)
IGFBP7 + MYBPC3	86.3 (79.5–93.2)
Creatinine + MYBPC3	86.3 (79.6–93.0)
DKK3 + MYBPC3	86.3 (79.6–93.0)
MYBPC3 + MR-proANP	86.3 (79.4–93.2)
MYBPC3 + Potassium	86.3 (79.5–93.1)
MYBPC3 + Glucose	86.3 (79.6–92.9)
FGF23 + Sodium	86.1 (79.1–93.1)
MYBPC3 + hsTnT	86.0 (79.1–92.9)
MYBPC3 + GDF15	85.8 (79.0–92.7)
Adiponectin + MYBPC3	85.8 (78.9–92.7)
IGFBP1 + MYBPC3	85.8 (78.9–92.6)
MR-proANP + Sodium	85.0 (77.3–92.6)

Ordered by decreasing AUC, based on patients with all biomarkers available.

ANGPT2, angiopoietin-2; BMP10, bone morphogenetic protein 10; CI, confidence interval; DKK3; Dickkopf-3; eGFR, estimated glomerular filtration rate; FABP3, fatty acid-binding protein 3; FGF23, fibroblast growth factor; GDF15, growth differentiation factor 15; IGF1 SD score, insulin-like growth Factor 1 standard deviation score; IGFBP1, insulin-like growth Factor 1 binding protein 1; insulin-like growth factor binding protein 7, IGFBP7; MYBPC3, cardiac myosin binding protein C; MR-proANP, MR-pro-atrial natriuretic peptide; MR-pro-adrenomedullin, MR-proADM; NT-proBNP, N-terminal pro-brain natriuretic peptide; sST2, soluble suppression of tumorigenicity 2; hsTnT, high sensitive troponin T.

### Association between seven novel biomarkers and clinical characteristics (Figure 1, Supplementary Figs. S1 and S2)

**Figure 1:**
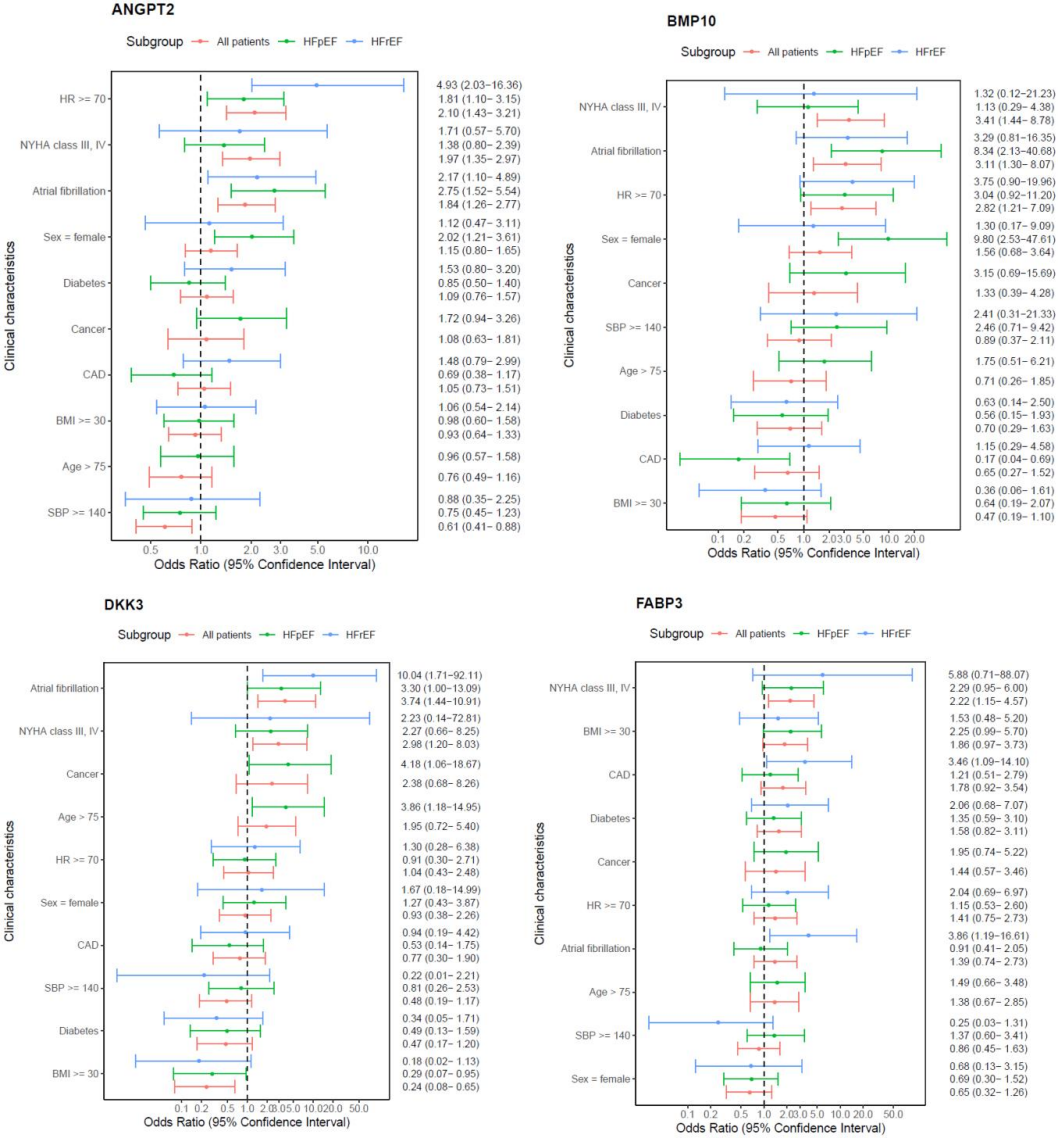

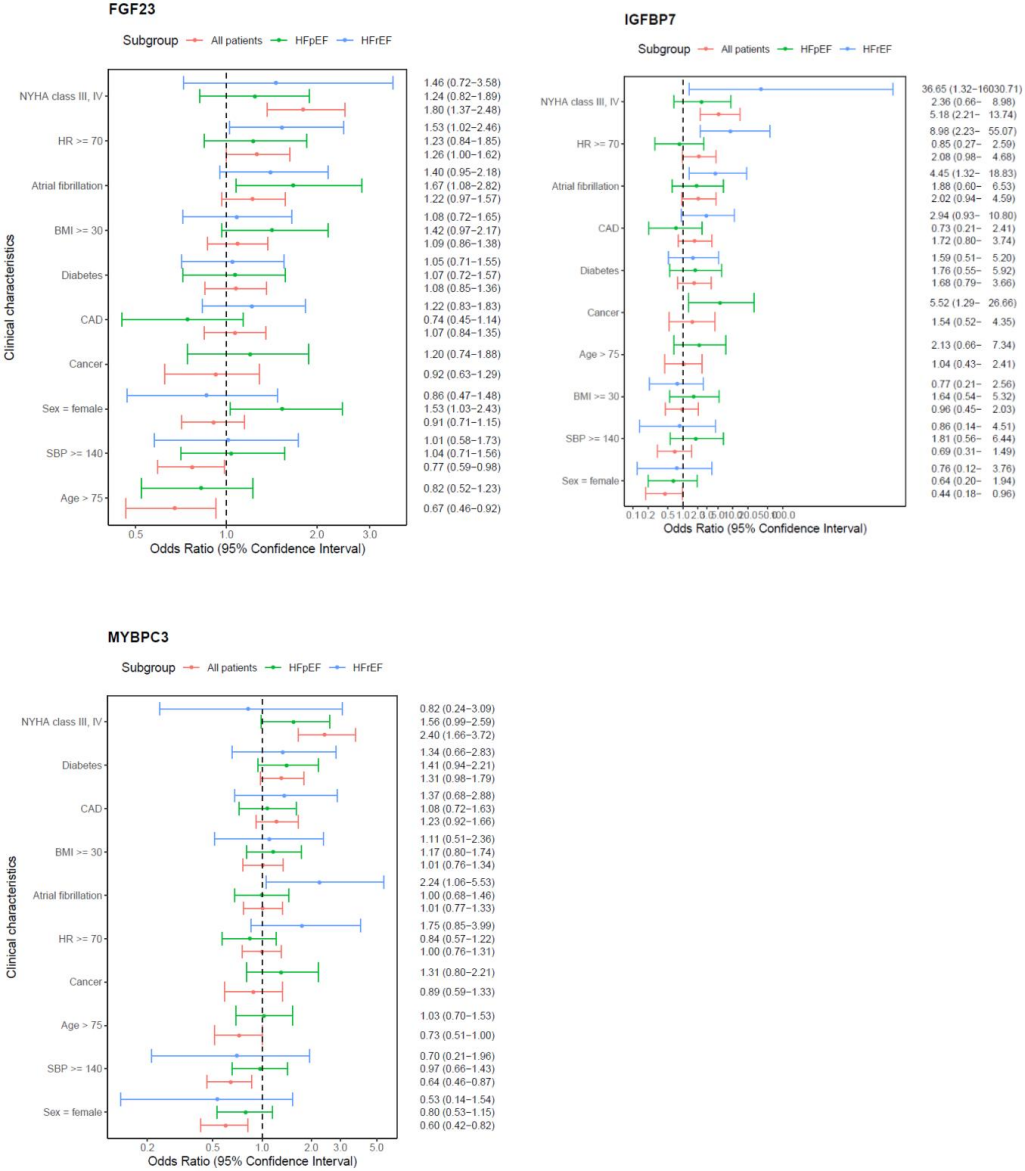
Associations between seven novel biomarkers and clinical characteristics in all, in heart failure with preserved and heart failure with reduced ejection fraction patients

Associations between the seven novel biomarkers and clinical characteristics in all and in HFpEF and HFrEF patients separately are depicted in *Figure 1*. When analysing both LVEF categories together, higher concentrations of all seven biomarkers were associated with worse functional class (NYHA Class III–IV vs I–II).

*ANGPT2*: A Higher ANGPT2 was associated with lower blood pressure (<140 mmHg), AFF, and higher heart rate (≥70 b.p.m.) in both HFpEF and HFrEF patients. In HFpEF, but not HFrEF, there was a potential association between ANGPT2 and female sex but with non-significant interaction (*P*_interaction_ = .275). In HFrEF, but not HFpEF, there was an association between ANGPT2 and lower eGFR (estimate −0.171 *P* = .035) (Supplementary Fig. S1).

*BMP10*: Similarly, higher BMP10 was in both HFpEF and HFrEF associated with AFF and higher heart rate and in HFpEF with female sex nominally but not significant in HFrEF (*P*_interaction_ = .099). In HFpEF BMP10 was also negatively associated with coronary artery disease (CAD).

*DKK3* was overall inversely associated with body mass index (< 30 kg/m^2^) and associated with AFF. Associations between DKK3 and clinical characteristic were similar between LVEF categories.

*FABP3* trended to overall be associated with obesity (≥30 kg/m^2^), with a more pronounced association in HFpEF. In HFrEF, but not HFpEF, FABP3 was nominally associated with AFF (*P*_interaction_ = .060) and CAD (*P*_interaction_ = .170). In HFpEF, FABP3 increased with higher NYHA class (*P* < .001) with similar but non-significant pattern in HFrEF, (*P* = .27) (Supplementary Fig. S2). Higher FGF23 was overall associated with younger age (≤75 years), higher heart rate and lower systolic blood pressure. In HFpEF FGF23 was higher with worse NYHA class (*P* = .005), associated with higher NT-proBNP and lower eGFR while only trends were observed in HFrEF (Supplementary Fig. S1 and S2).

*IGFBP7* was overall negatively associated with female sex and lower eGFR. In HFrEF, but not in HFpEF, IGFBP7 was associated with higher in heart rate (*P*_interaction_ = .016). In HFpEF, IGFBP7 was increased in higher NYHA class, (*P* = .062) (Supplementary Fig. S2) and with cancer.

*MYBPC3* was overall associated with higher NT-proBNP and male sex and lower systolic blood pressure, and borderline with younger age and diabetes. In HFrEF, but not HFpEF, there was an association with AFF (*P*_interaction_ = .073). Associations between all seven novel biomarkers and eGFR and NT-proBNP modelled by HF phenotype are presented in Supplementary Fig. S1 and NYHA class in Supplementary Fig. S2.

### Association between biomarkers and cardiac structure and function in heart failure with preserved ejection fraction (Table 5)

#### Associations with baseline Doppler echocardiographic parameters

Higher ANGPT2 was associated with lower left atrial strain and tricuspid annular plane systolic excursion (TAPSE) and with higher LV-GLS (expressed as a negative value) and indicating an association with impaired left atrial function and as well as right ventricular dysfunction. Higher BMP10 was associated with enlargement of the left atrium. Higher IGFBP7 and MYBPC3 were both associated with diastolic dysfunction expressed as higher *E*/*e*ʹ. Among traditional biomarkers, higher NT-proBNP and sST2 were negatively associated with left atrial strain indicating deteriorating left atrial function, further reflected by association between sST2 and increased LAVi. sST2 also predicted worsening systolic function by increased GLS. Gal-3 was associated with diastolic dysfunction reflected by higher *E*/*e*ʹ but also higher TAPSE, indicating preserved RV function. Higher MR-proADM was also with E/eʹ. Higher leptin was associated with lower LVEF and TnT with higher LV mass (*Table 5*).

**Table 5:** Association between biomarkers and myocardial structure and function in HFpEF

Biomarker	LVEF	LV-GLS	LVMI	LAVi	LA-GLS	TAPSE	*E*/*e*ʹ
	Beta coefficient Standard error; *P*						
ANGPT2 (ng/ml)	−1.37 1.01; 0.18	1.29 0.57; 0.03	−3.08 6.60; 0.64	4.20 2.51; 0.11	−5.03 1.36; <0.001	−1.03 0.49; 0.04	0.72 0.75; 0.34
BMP10 (ng/ml)	−1.95 2.92; 0.51	1.32 1.51; 0.39	−13.88 20.98; 0.51	23.78 7.10; 0.001	−4.33 3.71; 0.25	−1.19 1.31; 0.366	0.11 2.19; 0.96
DKK3 (ng/ml)	2.71 2.36; 0.26	0.58 1.34 0.67	−23.29 16.54; 0.17	10.00 6.53; 0.14	−2.25 3.29; 0.50	−1.77 1.14; 0.13	0.15 1.75; 0.93
FABP3 (ng/ml)	−2.95 1.78; 0.10	−0.28 1.04; 0.79	3.83 13.78; 0.78	6.01 5.37; 0.27	2.40 2.41; 0.32	1.33 0.87; 0.13	2.60 1.48; 0.08
FGF23 (pg/ml)	−0.84 0.83; 0.32	−0.04 0.44; 0.94	6.75 7.17; 0.35	3.23 2.83; 0.26	−0.01 1.20; 0.99	−0.2 0.38; 0.60	1.03 0.72; 0.16
IGFBP7 (ng/ml)	−1.48 2.42; 0.54	0.54 1.27; 0.67	−3.68 16.15; 0.82	11.70 6.03; 0.06	0.16 3.36; 0.96	−0.37 1.10; 0.74	4.09 1.70; 0.02
MYBPC3 (pg/ml)	−0.29 0.76; 0.70	0.25 0.44; 0.57	7.27 5.05; 0.16	1.63 2.06; 0.43	−0.61 1.12; 0.59	0.21 0.38; 0.59	1.36 0.50; 0.01
NT-proBNP [pg/ml)	−0.90 0.68; 0.19	0.57 0.39; 0.15	−0.42 3.82; 0.91	2.54 1.44; 0.09	−2.56 0.99; 0.01	−0.41 0.34; 0.23	0.43 0.49; 0.39
sST2 [µg/l)	−2.64 1.61; 0.11	1.86 0.75; 0.02	7.80 11.13; 0.49	9.70 4.07; 0.02	−5.62 1.86; <0.001	−1.30 0.66; 0.06	2.16 1.16; 0.07
GDF15 [pg/ml)	−1.24 1.17; 0.29	0.84 0.61; 0.17	11.29 6.65; 0.10	3.60 2.69; 0.19	0.40 1.50; 0.79	0.39 0.52; 0.46	1.35 0.82; 0.10
Galectin 3 [µg/l)	−1.00 1.74; 0.57	0.28 0.99; 0.77	15.94 10.28; 0.13	0.97 4.25; 0.82	3.43 2.29; 0.14	2.04 0.81; 0.02	3.48 1.23; 0.01
Copeptin [pmol/l)	−1.04 0.92; 0.26	0.54 0.49; 0.27	3.21 5.81; 0.58	1.23 2.31; 0.60	−0.43 1.22; 0.72	0.29 0.42; 0.49	0.78 0.67; 0.25
MR-proANP (pmol/l)	−1.60 1.40; 0.26	0.56 0.78; 0.48	−0.03 8.04; 1.00	5.17 3.04; 0.10	−1.45 1.97; 0.46	0.534 0.68; (0.435)	1.29 1.03; 0.22
MR-proADM (nmol/l)	−3.61 1.66; 0.04	1.26 0.97; 0.20	8.64 10.77; 0.43	7.71 4.08; 0.07	1.23 2.26; 0.59	0.23 0.84; 0.78	2.78 1.25; 0.03
hsTnT (pg/ml)	−0.38 0.90; 0.68	−0.19 0.53; 0.72	14.92 6.78; 0.04	0.83 2.92; 0.78	1.46 1.34; 0.28	0.51 0.45; 0.27	1.22 0.75; 0.11
IGF1 SD score	−0.89 0.95; 0.35	0.013 0.49; 0.98	−1.85 5.73; 0.75	−4.70 2.36; 0.06	1.68 1.26; 0.19	−0.12 0.44; 0.79	0.11 0.66; 0.87
IGFBP1	−0.39 0.93; 0.68	0.06 0.53; 0.91	−3.12 5.91; 0.60	0.06 2.36; 0.98	−0.27 1.42; 0.85	−0.17 0.45; 0.72	0.09 0.67; 0.89
Insulin	−2.99 1.11; 0.01	1.28 0.59; 0.03	5.66 6.69; 0.40	−0.71 2.69; 0.79	−2.19 1.66; 0.19	0.29 0.52; 0.58	−0.33 0.85; 0.70
Adiponectin	1.00 0.94; 0.30	−0.21 0.51; 0.68	−4.61 5.84; 0.44	−0.02 2.35; 0.99	−0.43 1.40; 0.76	−0.01 0.44; 0.99	−1.00 0.66; 0.14
Leptin	−1.86 0.63; <0.001	0.36 0.36; 0.32	5.16 3.95; 0.20	0.56 1.61; 0.73	−0.20 0.95; 0.83	0.27 0.31; 0.38	0.78 0.46; 0.10

ANGPT2, angiopoietin-2; BMP10, bone morphogenetic protein 10; DKK3, Dickkopf-3; eGFR, estimated glomerular filtration rate; FABP3, fatty acid-binding protein 3; FGF23, fibroblast growth factor; GDF15, growth differentiation factor 15; IGF1 SD score, insulin-like growth factor 1 standard deviation score; IGFBP1, insulin-like growth factor 1 binding protein; insulin-like growth factor binding protein 7, IGFBP7; LA-GLS, left atrial global longitudinal strain; LAVi, left atrial volume indexed; LV-GLS, left ventricle global longitudinal strain; LVEF, left ventricular ejection fraction; LVMI, left ventricular mass index; MYBPC3, cardiac myosin binding protein C; MR-proANP, MR-pro-atrial natriuretic peptide; MR-pro-adrenomedullin, MR-proADM; NT-proBNP, N-terminal pro-brain natriuretic peptide; sST2, soluble suppression of tumorigenicity 2; TAPSE, tricuspid annular plane systolic excursion; hsTnT, high sensitive troponin T.

#### Association between seven novel biomarkers and outcomes in heart failure with preserved fraction and Hear failure with reduced ejection fraction (Figure 2)

**Figure 2:**
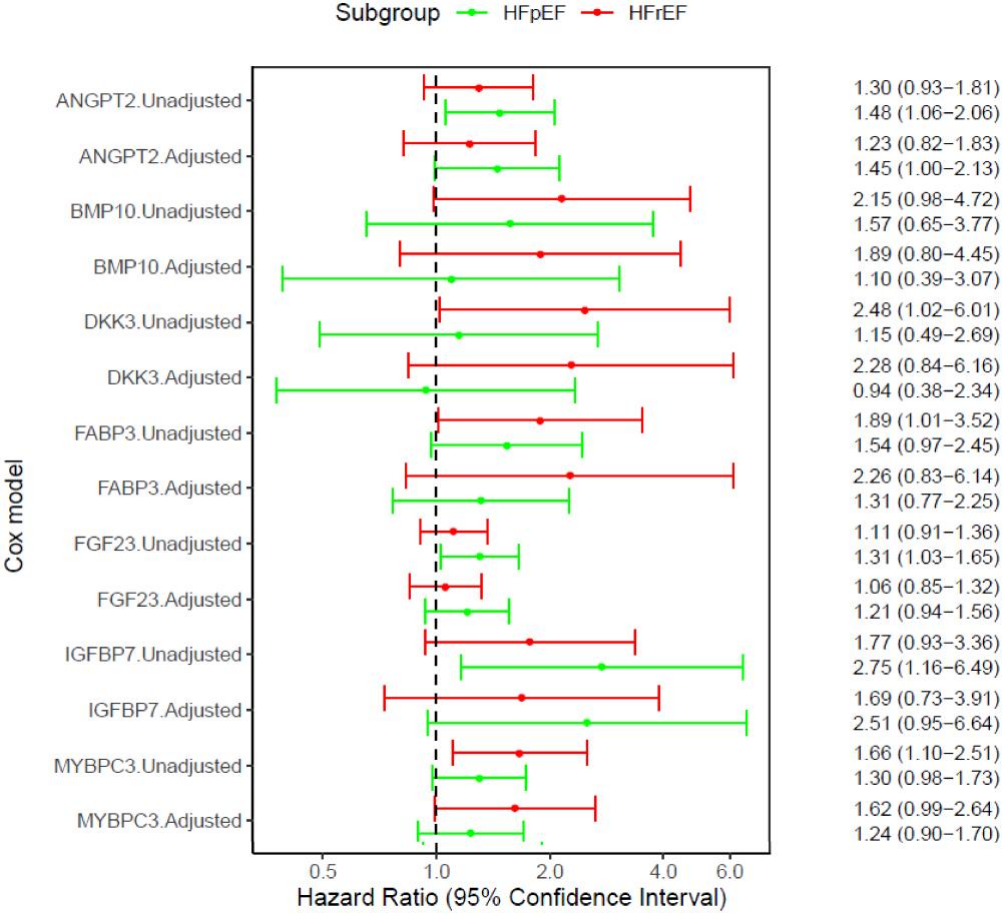
Association between seven novel biomarkers and outcome in heart failure with preserved and heart failure with reduced ejection fraction patients. Adjusted model includes covariates age, sex, and eGFR <60 ml/min/1.73 m^2^

Median follow-up times in HFpEF and HFrEF patients were 540 days (min; max 7; 1921) and 214 days (0; 1530), respectively. No patients were lost to follow-up. In HFpEF the composite outcome of all-cause death and HF hospitalization occurred in 31/76 patients, including 6 deaths, and in HFrEF patients, the composite outcome of death, LVAD implantation or HTx, occurred in 29/36 patients, including 18 deaths. Associations between biomarkers and the primary outcome in HFpEF and HFrEF respectively are presented in *Figure 2*.

In the overall cohort, higher concentrations of six of seven novel biomarkers were independently and significantly associated with the outcome, and the seventh, DKK3, nominally so. Independent predictors of outcome among the remaining (previously described) biomarkers were NT-proBNP, sST2, GDF-15, copeptin and IGF1 (Supplementary Table S2).

In HFpEF, the novel biomarkers, ANGPT2, FGF23 and IGFBP7 were in univariable analyses associated with the outcome. After adjusting for age, sex and eGFR <60 ml/min/1.73 m^2^ none of the novel biomarkers remain statistically significant but ANGPT2 [HR 1.45 (95% CI 1.00–2.13); *P* = .05] and IGFBP7 [HR 2.51 (95% CI 0.95–6.64); *P* = .06) were borderline (*Figure 2*). NT-proBNP was numerically associated with outcome but did not reach statistical significance [HR 1.28 (95% CI 0.96 1.71); *P* = .10].

In HFrEF, higher DKK3, FABP3 and MYBPC3 were associated with outcome in univariable analyses. MYBPC3 [HR 1.62 (95% CI 0.99–2.64); *P* = .06] remained borderline significant in the adjusted model (Figure 2). Among remaining biomarkers significant or borderline independent predictors were IGF1 [HR 0.64 (95% CI 0.41–0.99); *P* = .04], insulin [HR 0.75 (95% CI 0.55–1.01); *P* = .06] and copeptin [HR 1.91 (95% CI 0.95–3.81); *P* = .07]. NT-proBNP was not prognostic [HR 1.20 (95% CI 0.88–1.63); *P* = .25].

## Discussion

We present comprehensive data on seven novel biomarker assays, potentially reflecting differences between HFrEF and HFpEF in underlying pathophysiology and outcomes. Six (not FABP3) of the seven biomarkers were higher in HFrEF vs HFpEF and all were associated with worse NYHA class and eGFR in both LVEF categories. Of the seven biomarkers, MYBPC3 and FGF23 had the highest discriminative power between HFrEF and HFpEF. Six of the novel biomarkers, with a trend for the seventh (DKK3), were associated with the outcome, with the strongest signal in HFpEF for ANGPT2 and IGFBP7 and in HFrEF for MYBPC3.

Our findings are summarized in Supplementary Table S3 and graphical abstract. FGF23, higher in HFrEF vs HFpEF, performed well as a discriminator contrasting to previous reports in incident HFrEF vs HFpEF.^12,13^ We found that some biomarkers, such as DKK3 and FABP3 displayed ambiguous roles in HF pathophysiology with no clear distinction between LVEF categories which may be due to the small sample size. In HF FABP3 has previously been demonstrated as a marker of acute cellular damage and cardiac functional impairment and DKK3 of declining kidney function. We showed both biomarkers hold valuable information on HF severity and prognosis. Circulating biomarkers may act both as markers but also as drivers of disease. Our data is consistent with data that ANGPT2 contributes to microvascular injury^14^ and subsequent microvascular dysfunction, prominent in HFpEF. This biomarker carries important information in the initiation and progression of HF, perhaps more so in HFpEF compared with HFrEF.^7,8^ Also FGF23 may have direct implications in HFpEF, especially in atrial fibrillation^15^ and kidney dysfunction.^16^ Biomarkers were overall higher in HFrEF vs HFpEF but displaying patterns in each LVEF category indicative of muscle injury and myopathy in HFrEF and endothelial and oxidative stress in HFpEF. These findings support primary cardiomyocyte injury as a driver of HFrEF and systemic inflammation as a driver of HFpEF.

### Biomarkers—how to use them

Novel biomarkers and new improved assays used in statistical models such as cluster analysis or machine learning models may provide more specific and comprehensive knowledge on diagnosis and prognosis and inform on underlying disease mechanisms. Based on biomarkers quantified by the same assay as in the present study, Fabritz *et al*.^17^ have in cluster analysis of atrial fibrillation patients identified BMP10, IGFBP7, ANGPT2, GDF15, and NT-proBNP associated with the highest risk and with highest prevalence of HF. Specific biomarkers have differed between studies, but they imply overall unified patterns in activated pathways differing by HF phenotype. Heart failure with reduced ejection fraction is on a molecular level characterized by myocardial metabolism, cellular proliferation and stretch, related to lower LVEF^18–20^ that successfully have been targeted by blocking neurohormonal activation. Heart failure with preserved ejection fraction patients demonstrate endothelial microvascular dysfunction and inflammation, endothelial dysfunction, cell migration, immune activation and extracellular matrix reorganization.^21–26^ In HFpEF as in HFrEF, NT-proBNP reflecting neurohormonal activation is prognostic, but in HFpEF biomarkers reflecting inflammation and endothelial dysfunction may outperform NT-proBNP as prognostic markers.^23^ Therapies suppressing inflammation, oxidative stress and restoring cellular metabolism may be attractive and are presently investigated by the use of MPO-inhibitors^27^ and IL-6 inhibitors (NCT05636176). Finding future treatment targets is crucial, such as restoring the depressed MYBPC3 phosphorylation through sildenafil or myeloperoxidase inhibition to improve relaxation defects in cardiomyocytes and improving cardiac dysfunction^28,29^ or IGFBP7 vaccination therapy.^30^

#### Limitations

These are exploratory analyses performed in two smaller cohorts with limited statistical power and with no formal power calculation and should therefore be interpreted with caution as hypothesis-generating.

The small sample size limits statistical models, thus several highly relevant covariates such as eGFR and NT-proBNP did not fit into the models. As an example, the well-established biomarker NT-proBNP was prognostic when analysed in the overall population but did not reach statistical significance when analysed in HFpEF and HFrEF separately. The novel biomarkers were measured using non-commercial assays that were at various stages of analytical validation. The two study protocols differed in inclusion criteria and outcomes, due to the different HF populations enrolled. Blood sampling was performed according to the same procedures. According to the protocol in the KaRen HFpEF study, the definition of HFpEF reflected the concept at the study start, structural heart disease or diastolic dysfunction was not required as the study was designed prior to the present guidelines; however, 94% of the study population did comply with the present criteria of HFpEF by ESC 2021 guidelines.^1^

## Conclusion

This hypothesis-generating study of seven novel biomarkers provides further information on HF disease mechanisms exploring underlying pathophysiology that differs with LVEF categories. This information will contribute to better phenotyping of HF, paving the way for development of novel therapies and personalizing treatment and potentially allow clinicians to perform biomarker-guided therapy specifically designed for individual patients.

## Supplementary Material

xvaf011_Supplementary_Data

## Declarations

### Data Availability

All data are incorporated into the article and its online supplementary material.

### Pre-registered Clinical Trial Number

None supplied.
