# Supplementary material for: Novel biomarkers distinguish heart failure with preserved vs reduced ejection fraction
Source: ESC Heart Fail. 2026 Jan 8;13(3):xvaf011. doi: 10.1093/eschf/xvaf011 (PMC13228998; doi:10.1093/eschf/xvaf011)
Supplement: xvaf011_Supplementary_Data [file xvaf011_supplementary_data.zip › Supplemental Table 2 22MAY2025.docx]

**Supplemental Table 2.** Associations between biomarkers and outcome in overall cohort including both HFpEF and HFrEF patient. Biomarkers are presented as hazard ratio (HR) 95% confidence interval (CI) and p-value.

| Biomarker | Unadjusted | p-value | Adjusted Model  (age, sex, eGFR <60 ) | p-value |
| --- | --- | --- | --- | --- |
| ANGPT2 [ng/mL] | 1.53 1.22 1.92 | <0.001 | 1.512 1.195 1.914 | <0.001 |
| BMP10 [ng/mL] | 2.43 1.39 4.24 | <0.001 | 2.21 1.24 3.95 | 0.01 |
| DKK3 [ng/mL] | 1.78 1.00 3.17 | 0.05 | 1.73 0.94 3.16 | 0.07 |
| FABP3 [ng/mL] | 1.61 1.14 2.27 | 0.01 | 1.59 1.03 2.44 | 0.04 |
| FGF23 [pg/ml] | 1.31 1.14 1.50 | <0.001 | 1.24 1.07 1.44 | <0.001 |
| IGFBP7 [ng/ml] | 2.68 1.62 4.41 | <0.001 | 2.49 1.40 4.46 | 0.001 |
| MYBPC3 [pg/ml] | 1.55 1.27 1.89 | <0.001 | 1.512 1.196 1.911 | <0.001 |
|  |  |  |  |  |
| NTproBNP [pg/ml] | 1.43 1.21 1.70 | <0.001 | 1.377 1.149 1.65 | <0.001 |
| sST2 [µg/L] | 2.15 1.51 - 3.07 | <0.001 | 2.04 1.39 - 3.00 | <0.001 |
| GDF-15 [pg/ml] | 1.42 1.14 1.77 | <0.001 | 1.38 1.06 1.79 | 0.02 |
| Galectin 3 [µg/L] | 1.20 0.80 - 1.81 | 0.37 | 0.88 0.52 - 1.50 | 0.65 |
| Copeptin [pmol/L ] | 1.65 1.30 2.09 | <0.001 | 1.61 1.19 2.18 | <0.001 |
| MR-proANP [pmol/L] | 1.73 1.23 2.43 | <0.001 | 1.62 1.13 2.32 | 0.01 |
| MR-proADM [nmol/L] | 1.62 1.12 2.35 | 0.01 | 1.53 0.97 2.42 | 0.07 |
| hsTnT [pg/ml] | 1.40 1.14 1.71 | <0.001 | 1.32 1.05 1.66 | 0.02 |
| IGF1 | 0.58 0.42 0.80 | <0.001 | 0.60 0.44 0.82 | <0.001 |
| IGFBP1 | 1.16 0.90 1.48 | 0.25 | 1.15 0.89 1.49 | 0.28 |
| Insulin | 0.95 0.73 1.23 | 0.70 | 0.89 0.68 1.16 | 0.40 |
| Adiponectin | 1.07 0.85 1.35 | 0.56 | 1.19 0.92 1.54 | 0.17 |
| Leptin | 1.01 0.86 1.19 | 0.90 | 0.96 0.81 1.14 | 0.64 |

Abbreviations: ANGPT2=Angiopoietin-2; BMP10=Bone morphogenetic protein 10; DKK3=Dickkopf-3; eGFR=estimated glomerular filtration rate; FABP3=Fatty acid-binding protein 3; FGF23=Fibroblast growth factor; GDF15=Growth differentiation factor 15; HOMA-IR=Homeostatic Model Assessment-Insulin Resistance; IGF1=Insulin-like Growth Factor 1; IGFBP1=Insulin-like Growth Factor 1 binding protein 1; Insulin-like growth factor binding protein 7=IGFBP7; MYBPC3=Cardiac myosin binding protein C; MRproANP=MR-pro-atrial natriuretic peptide; MR-proADM=MR-pro-adrenomedullin; NT-proBNP=N-terminal pro-brain natriuretic peptide; sST2=Soluble suppression of tumorigenecity 2; hsTnT=high sensitive troponin T
