# Supplementary material for: Novel biomarkers distinguish heart failure with preserved vs reduced ejection fraction
Source: ESC Heart Fail. 2026 Jan 8;13(3):xvaf011. doi: 10.1093/eschf/xvaf011 (PMC13228998; doi:10.1093/eschf/xvaf011)
Supplement: xvaf011_Supplementary_Data [file xvaf011_supplementary_data.zip › Supplemental Table 3 08Oct2025.docx]

**Supplemental Table 3.** Novel biomarker assays, mode of action, discriminating power between HFpEF and HFrEF and interpretation of results.


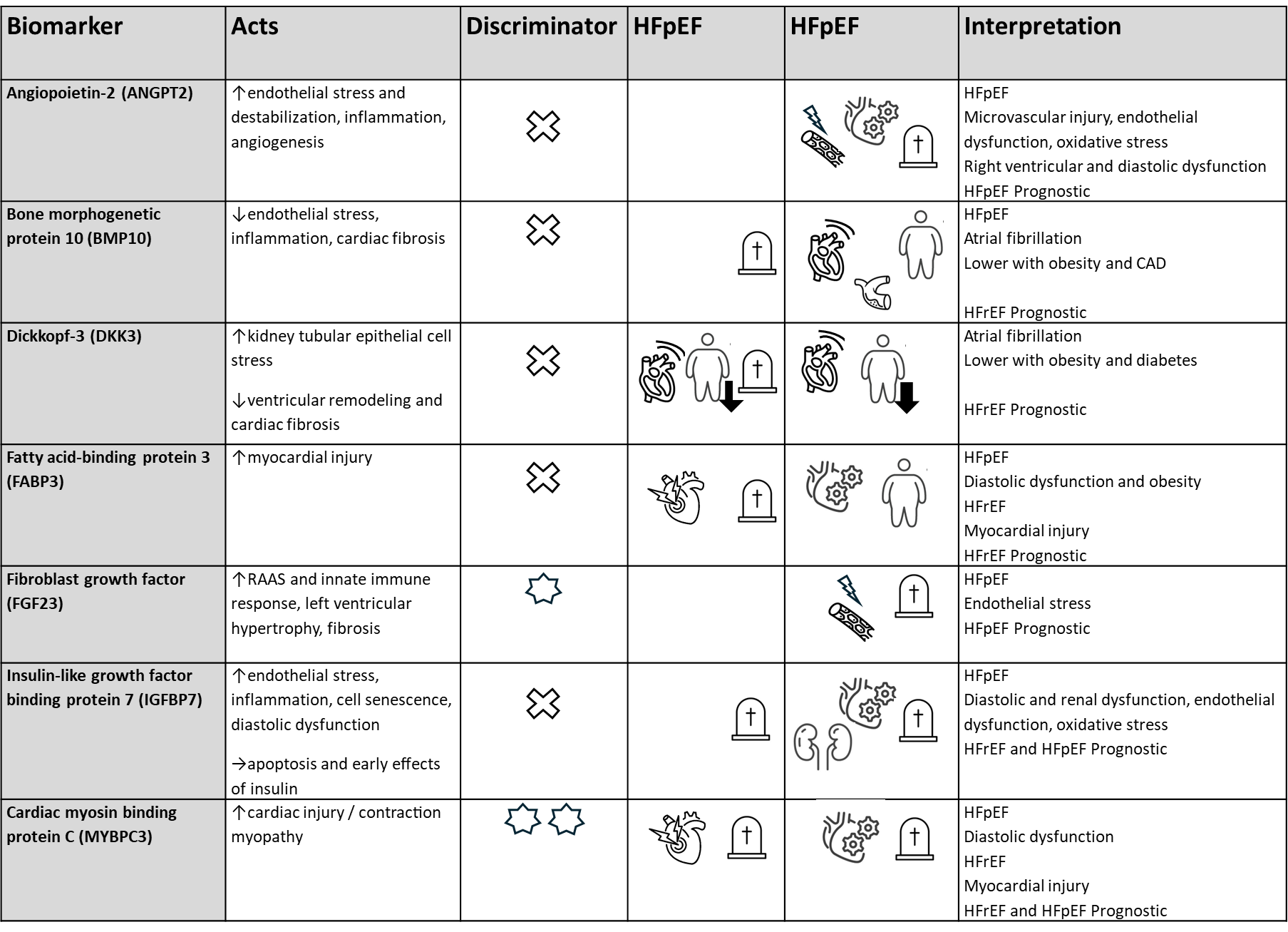


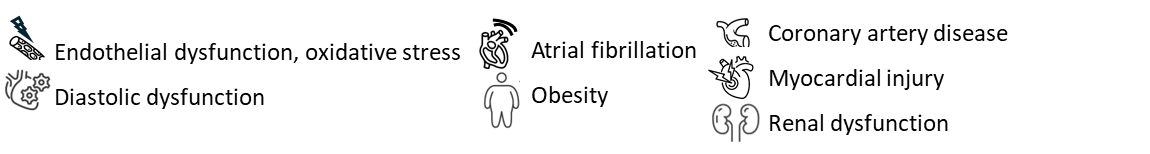


*iStock.com/Artist's Magnilion/leremy/VikiVector/*
