# Supplementary material for: Novel biomarkers distinguish heart failure with preserved vs reduced ejection fraction
Source: ESC Heart Fail. 2026 Jan 8;13(3):xvaf011. doi: 10.1093/eschf/xvaf011 (PMC13228998; doi:10.1093/eschf/xvaf011)
Supplement: xvaf011_Supplementary_Data [file xvaf011_supplementary_data.zip › Supplemental Figure 1 22MAY2025.docx]

Supplemental Figure 1.

Correlation between eGFR and biomarkers in HFpEF and HFrEF expressed as estimate, standard error and p-value.


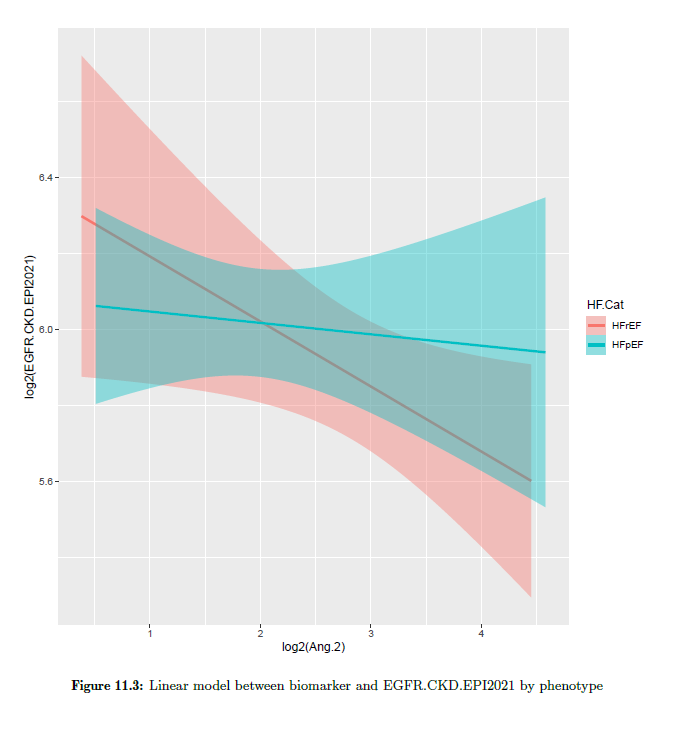

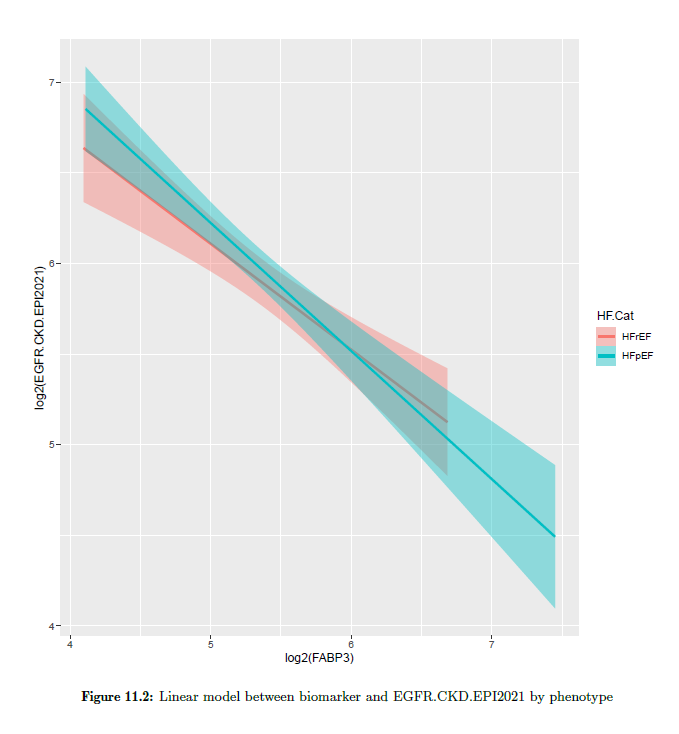


HFrEF -0.171 0.078 0.035 HFrEF -0.584 0.102 <0.001

HFpEF -0.03 0.074 0.685 HFpEF -0.707 0.089 <0.001


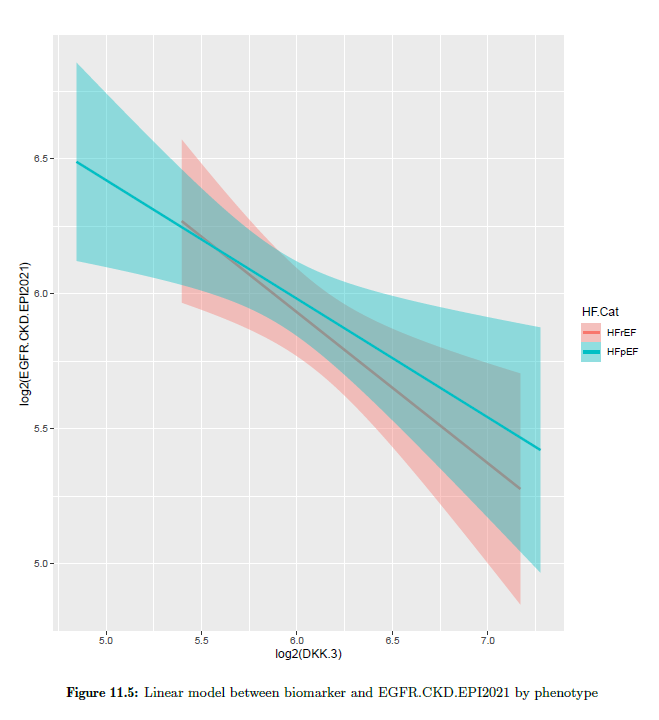

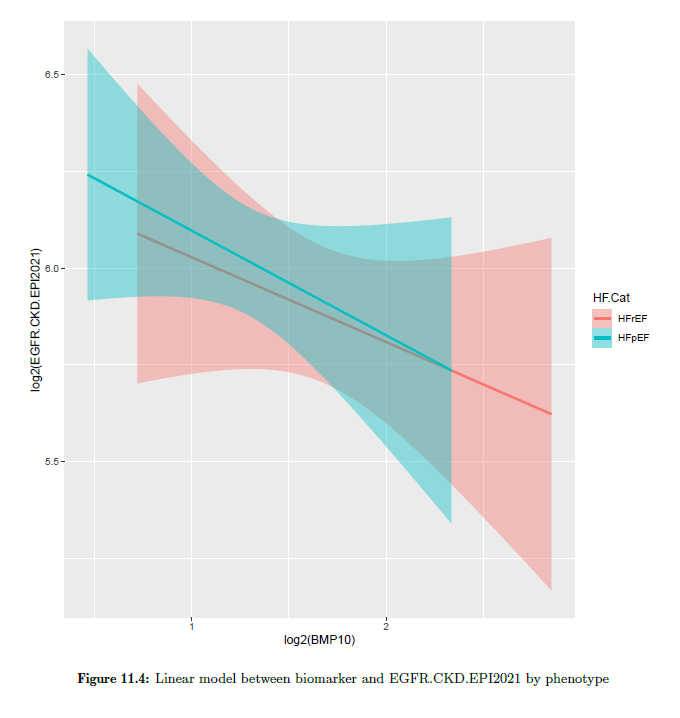


HFrEF -0.559 0.181 0.004 HFrEF -0.219 0.177 0.224

HFpEF -0.439 0.16 0.008 HFpEF -0.271 0.178 0.133


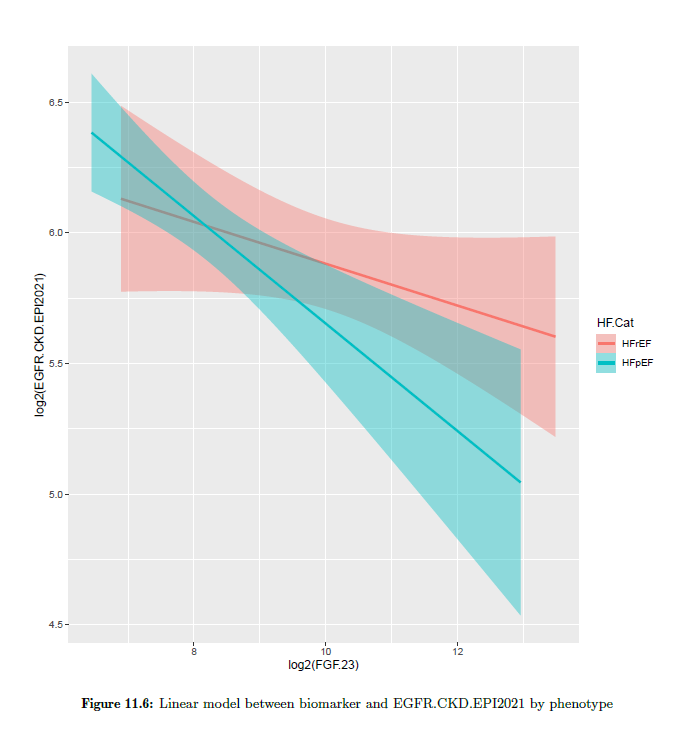

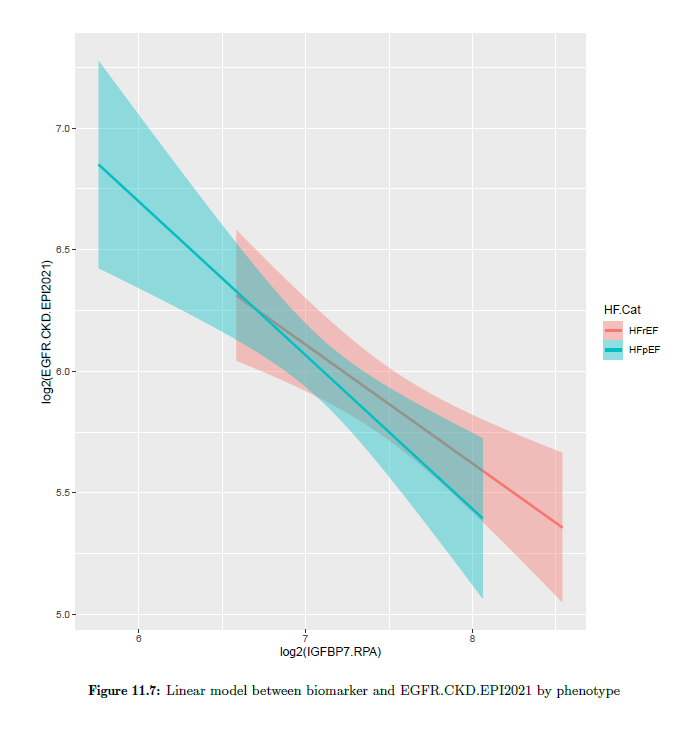


HFrEF -0.08 0.049 0.109 HFrEF -0.489 0.124 <0.001

HFpEF -0.206 0.052 <0.001 HFpEF -0.632 0.155 <0.001


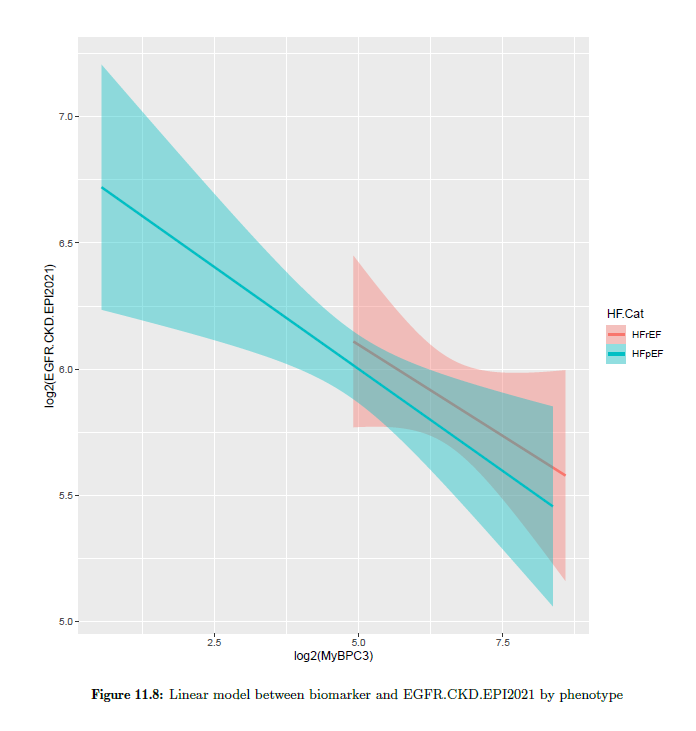

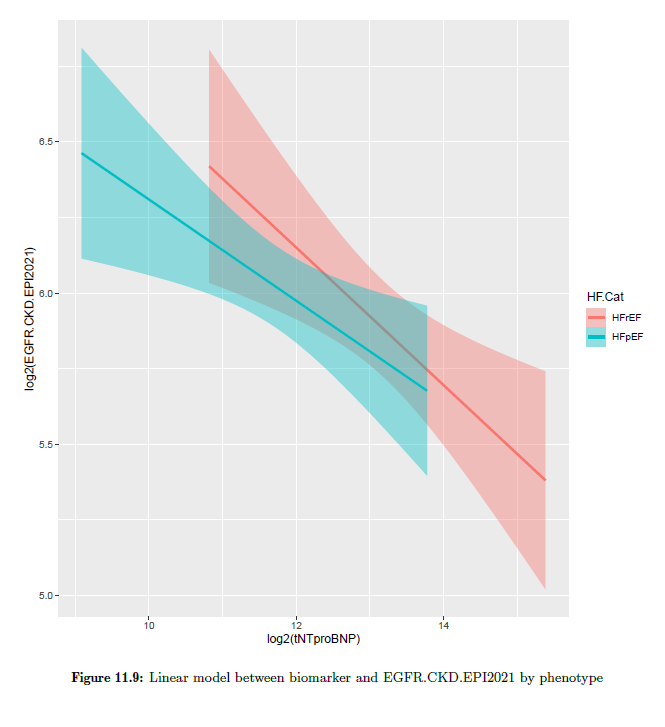


HFrEF -0.144 0.09 0.118 HFrEF -0.227 0.073 0.004

HFpEF -0.162 0.054 0.004 HFpEF -0.167 0.061 0.007

Correlation between NT-proBNP and biomarkers in HFpEF and HFrEF expressed as estimate, standard error and p-value.


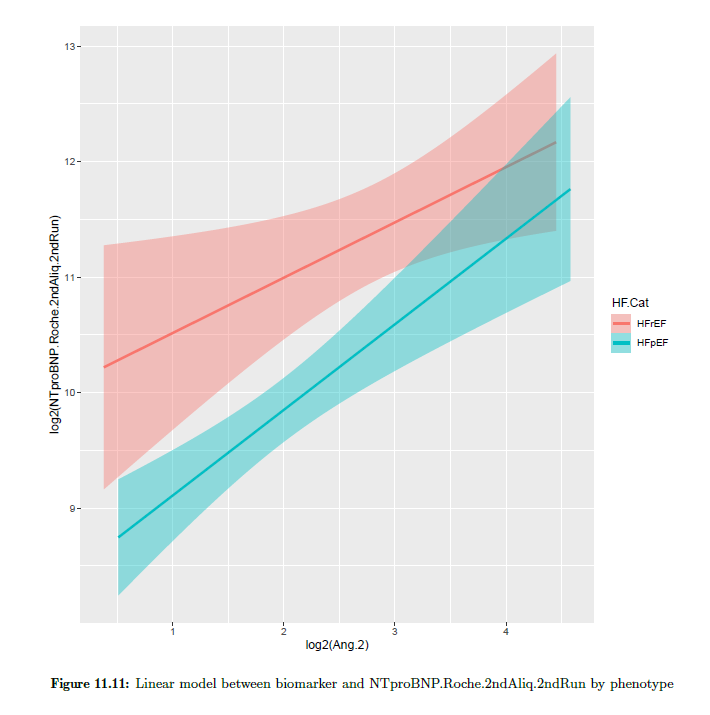

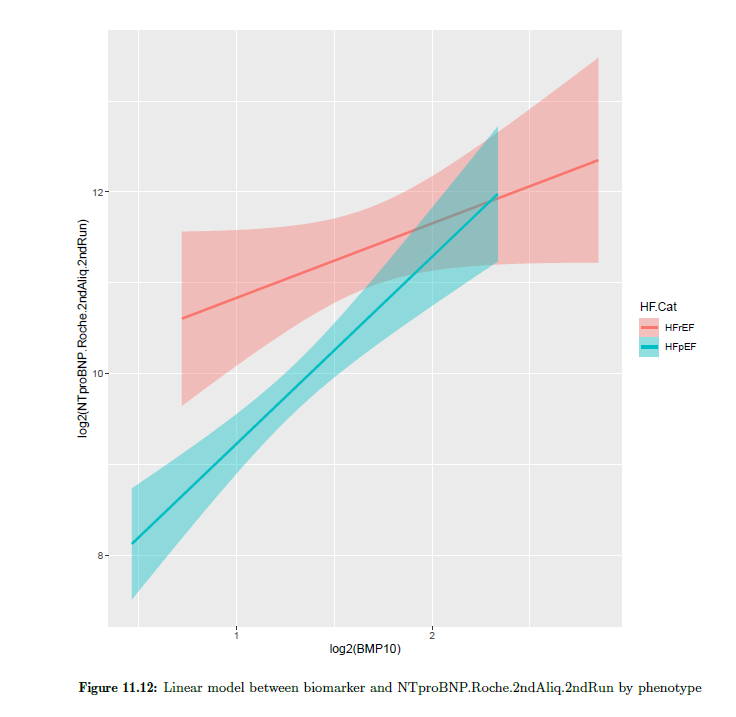


HFrEF 0.479 0.195 0.019 HFrEF 0.819 0.438 0.07

HFpEF 0.742 0.144 <0.001 HFpEF 2.058 0.334 <0.001


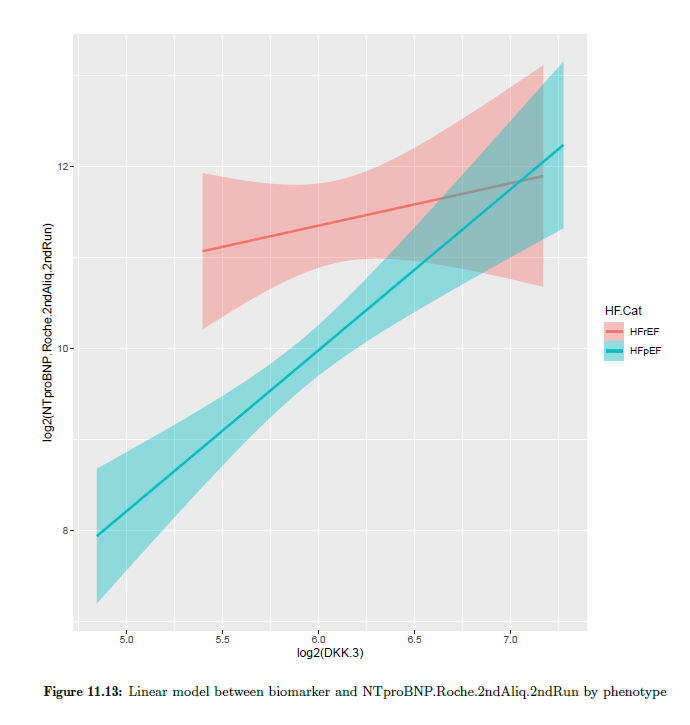

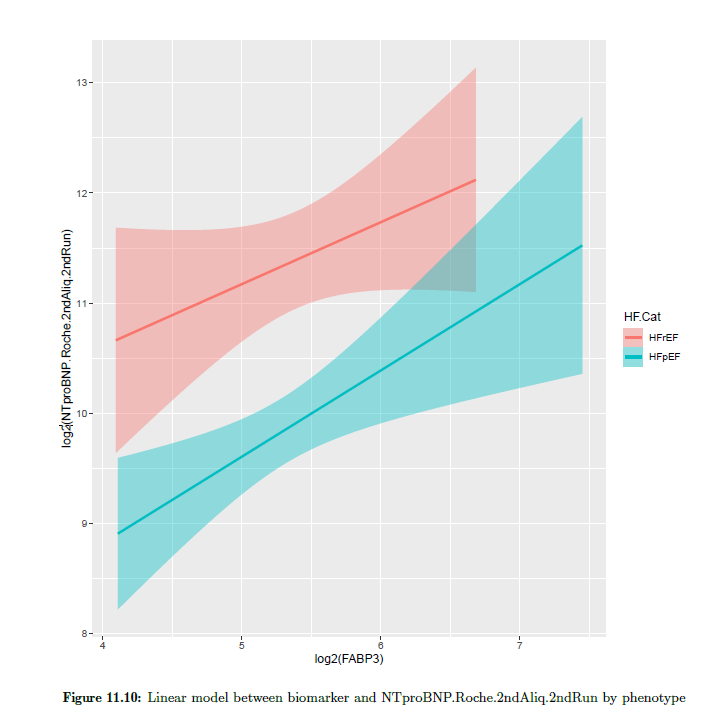


HFrEF 0.465 0.515 0.373 HFrEF 0.563 0.35 0.117

HFpEF 1.767 0.322 <0.001 HFpEF 0.784 0.261 0.004


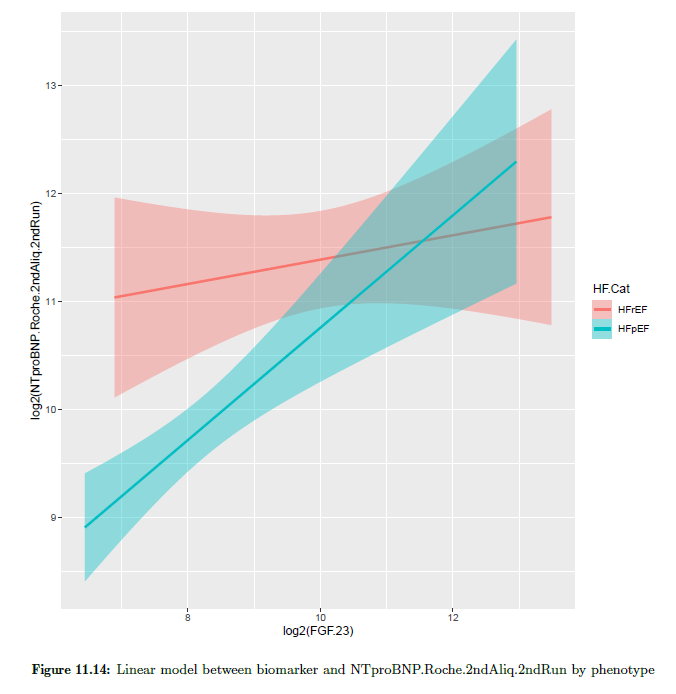

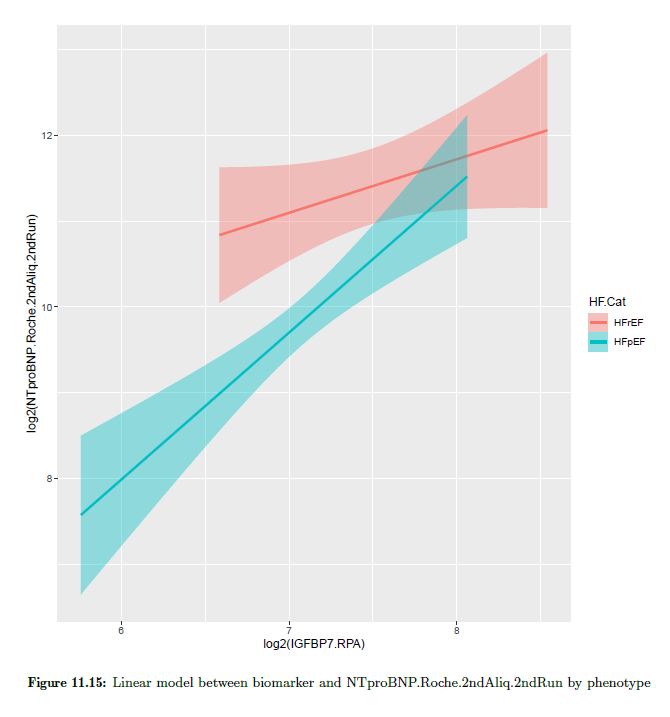


HFrEF 0.113 0.127 0.38 HFrEF 0.625 0.365 0.096

HFpEF 0.521 0.116 <0.001 HFpEF 1.714 0.336 <0.001


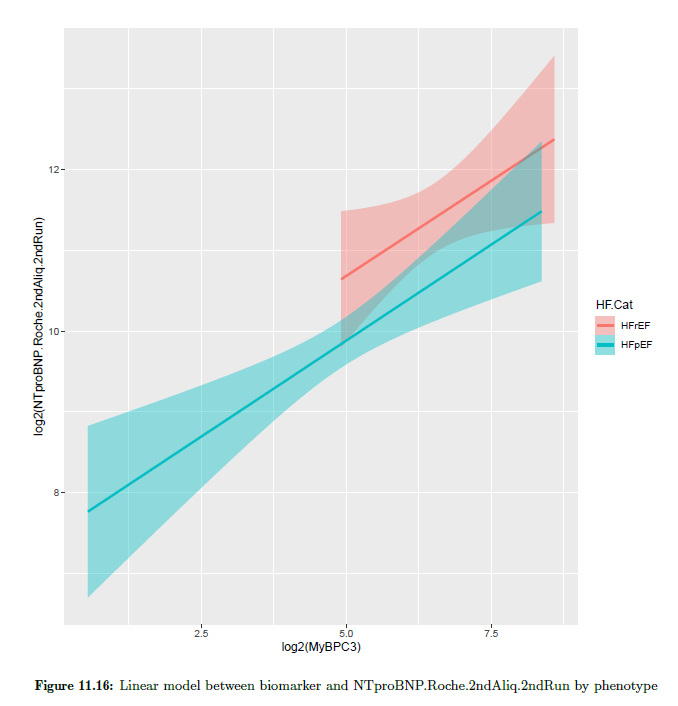


HFrEF 0.471 0.223 0.042

HFpEF 0.475 0.117 <0.001
