# Supplementary material for: Novel biomarkers distinguish heart failure with preserved vs reduced ejection fraction
Source: ESC Heart Fail. 2026 Jan 8;13(3):xvaf011. doi: 10.1093/eschf/xvaf011 (PMC13228998; doi:10.1093/eschf/xvaf011)
Supplement: xvaf011_Supplementary_Data [file xvaf011_supplementary_data.zip › Supplemental Material Biomarkers 22MAY2025.docx]

**Supplemental text**

*(I) Angiopoietin-2 (ANGPT2, UniProt ID O15123),* a Tie-2 receptor agonist and an endothelial growth factor mediating angiogenesis released in response to endothelial stress and inflammation. It is a promoter of cell infiltration inducing endothelial cell apoptosis (1). The ANGPT2 protein has been associated with incident and worsening of HF (2), increased cardiac size and lower function and clinical outcomes (3) and features common in HFpEF such as AF, diabetes and female sex (4-6).

*(II) Bone morphogenetic protein 10 (BMP10, UniProt ID O95393)*, a protein belonging to the transforming growth factor-beta (TGF-β) superfamily. BMP10 is predominantly expressed in the right atrium (7, 8)., plays a role in heart development and protection and is an endothelial activator with ability to inhibit endothelial cell apoptosis (9). In patients HF BMP10 is higher, associated with atrial remodeling and outcomes (10, 11) and has been suggested to enhance contractile vascular smooth muscle cells (VSMCs) (12) promote cardiac hypertrophy, remodeling and systolic dysfunction (13).

*(III) Dickkopf-3 (DKK3, UniProt ID Q9UBP4)*, a secreted glycoprotein of the Dickkopf family, regulator of Wnt signaling and cell development in many tissues such as bone tissue, the neural epithelium, the heart, and immune response. DKK3 is highly expressed in cardiac tissue implying a value as a cardiac-specific biomarker (7). DKK3 has in HF models been ascribed cardioprotective effects on ventricular remodeling (14). But in patients with HF higher urinary DKK3 is associated with declining kidney function (15), sarcopenia and lowering of DKK3 increased physical performance (16). Despite associated with co-existing conditions with HF, DKK3s has been found to lack prognostic impact (7), possibly explained by age or course of HF (17).

*(IV) Fatty acid-binding protein 3 (FABP3, UniProt ID P05413)* or heart FABP (H-FABP) is a cytoplasmic protein participating in lipid transportation of transport long-chain polyunsaturated fatty acids from cell membranes to the mitochondria in the cardiomyocytes. FABP3 is rapidly released from cardiomyocytes upon myocardial injury and may act as a marker of cellular damage in the acute stage, but also myocardial functional impairment in the chronic settings (18). FABP3 is suggested as both promotor and inhibitor of myocyte proliferation and apoptosis, and loss may lead to progression of hypertrophy and HF (19, 20). In HF FABP3 is a prognostic predictor, and in HFrEF associated with higher NYHA functional class, lower physical performance and LVEF (21-23).

*(V) Fibroblast growth factor (FGF23, UniProt ID Q9GZV9)* is a hormone secreted predominantly by osteoblasts but also by the myocardium and regulate serum phosphate, parathyroid hormone, vitamin D and iron metabolism (24). FGF23 stimulates cardiac hypertrophy, myocardial fibrosis with subsequent diastolic dysfunction via upregulation of TGF-β and β -catenin (25). In HFrEF FGF23 is proposed as a marker of congestion associated with inflammation and remodeling pathways (26, 27). In HF increased FGF23 may contribute to cardiac risk through direct induction of cardiac remodeling and vascular calcification (28) and is associated with outcomes across the LVEF spectrum (29, 30).

*(VI) Insulin-like growth factor binding protein 7 (IGFBP7, UniProt Q16270),* also called IGFBP-related protein-1 or angiomodulin, is a secretory glycoprotein expressed in various tissues including the heart, vascular endothelial cells, and fibroblasts. It binds to insulin-like growth factor-I (IGF-I) regulating cell death and early effects of insulin action.

IGFBP7 exacerbates endothelial cell injury by activating the NFκB signaling (31) and promote cardiac fibrosis through the Htra3-TGF-β-IGFBP7 pathway (32), core features and disease drivers in HFpEF (33). IGFBP7 was initially investigated as a marker of diastolic dysfunction highlighted in HFpEF (34-36). In HFrEF we and others have shown that IGFBP7 declines with improved hemodynamics after LVAD and heart transplantation (35, 37). IGFBP7 may have a direct role in the development of HF (38, 39) and is a strong predictor of cardiovascular events and mortality in HFrEF and HFpEF (35, 40-43).

*(VII) Cardiac myosin binding protein C (MYBPC3, cMyBP-C, UniProt ID Q14896),* encoded by the cardiac specific MYBPC3 gene, is a contractile protein that interacts with myosin, titin and actin and regulates cardiac muscle contraction. In HF, decreased MYBPC3 protein phosphorylation may (44) may contribute to a compromised contractile function and diastolic dysfunction (45, 46). Further, mutations in the MYBPC3 gene are one of the most frequent triggers of hypertrophic cardiomyopathy (47, 48). Circulating MYBPC3 has been suggested as a marker for HF diagnosis, severity and prognosis (49, 50) and myocardial injury (51).

**References**

1. Fagiani E, Christofori G. Angiopoietins in angiogenesis. *Cancer Lett* 2013;**328**:18-26.

2. Peplinski BS, Houston BA, Bluemke DA, Kawut SM, Kolb TM, Kronmal RA, et al. Associations of Angiopoietins With Heart Failure Incidence and Severity. *J Card Fail* 2021;**27**:786-95.

3. Harrington J, Nixon AB, Daubert MA, Yow E, Januzzi J, Fiuzat M, et al. Circulating Angiokines Are Associated With Reverse Remodeling and Outcomes in Chronic Heart Failure. *J Card Fail* 2023;**29**:896-906.

4. Benz AP, Hijazi Z, Lindbäck J, Connolly SJ, Eikelboom JW, Kastner P, et al. Plasma angiopoietin-2 and its association with heart failure in patients with atrial fibrillation. *Europace* 2023;**25**.

5. Florijn BW, Valstar GB, Duijs J, Menken R, Cramer MJ, Teske AJ, et al. Sex-specific microRNAs in women with diabetes and left ventricular diastolic dysfunction or HFpEF associate with microvascular injury. *Sci Rep* 2020;**10**:13945.

6. Chua W, Cardoso VR, Guasch E, Sinner MF, Al-Taie C, Brady P, et al. An angiopoietin 2, FGF23, and BMP10 biomarker signature differentiates atrial fibrillation from other concomitant cardiovascular conditions. *Sci Rep* 2023;**13**:16743.

7. Piek A, Suthahar N, Voors AA, de Boer RA, Sillje HHW. A combined bioinformatics, experimental and clinical approach to identify novel cardiac-specific heart failure biomarkers: is Dickkopf-3 (DKK3) a possible candidate? *Eur J Heart Fail* 2020;**22**:2065-74.

8. Neuhaus H, Rosen V, Thies RS. Heart specific expression of mouse BMP-10 a novel member of the TGF-beta superfamily. *Mechanisms of development* 1999;**80**:181-4.

9. Wang X, Sun H, Yu H, Du B, Fan Q, Jia B, et al. Bone morphogenetic protein 10, a rising star in the field of diabetes and cardiovascular disease. *J Cell Mol Med* 2024;**28**:e18324.

10. Hennings E, Blum S, Aeschbacher S, Coslovsky M, Knecht S, Eken C, et al. Bone Morphogenetic Protein 10-A Novel Biomarker to Predict Adverse Outcomes in Patients With Atrial Fibrillation. *J Am Heart Assoc* 2023;**12**:e028255.

11. Ceelen DCH, Bracun V, van Essen BJ, Voors AA, de Boer RA, Ter Maaten JM, et al. Circulating bone morphogenetic protein 10 as a novel marker of atrial stress and remodelling in heart failure. *Heart* 2024.

12. Wang L, Rice M, Swist S, Kubin T, Wu F, Wang S, et al. BMP9 and BMP10 Act Directly on Vascular Smooth Muscle Cells for Generation and Maintenance of the Contractile State. *Circulation* 2021;**143**:1394-410.

13. Bouvard C, Tu L, Rossi M, Desroches-Castan A, Berrebeh N, Helfer E, et al. Different cardiovascular and pulmonary phenotypes for single- and double-knock-out mice deficient in BMP9 and BMP10. *Cardiovasc Res* 2021.

14. Bao MW, Cai Z, Zhang XJ, Li L, Liu X, Wan N, et al. Dickkopf-3 protects against cardiac dysfunction and ventricular remodelling following myocardial infarction. *Basic Res Cardiol* 2015;**110**:25.

15. Pieper D, Sandek A, Schäfer AK, Dihazi H, Dihazi GH, Leha A, et al. Urinary Dickkopf-3 as a Potential Marker for Estimated Glomerular Filtration Rate Decline in Patients With Heart Failure. *J Am Heart Assoc* 2024;**13**:e036637.

16. Karim A, Muhammad T, Shah I, Khan J, Qaisar R. A multistrain probiotic reduces sarcopenia by modulating Wnt signaling biomarkers in patients with chronic heart failure. *J Cardiol* 2022;**80**:449-55.

17. Yin J, Yang L, Xie Y, Liu Y, Li S, Yang W, et al. Dkk3 dependent transcriptional regulation controls age related skeletal muscle atrophy. *Nat Commun* 2018;**9**:1752.

18. Rezar R, Jirak P, Gschwandtner M, Derler R, Felder TK, Haslinger M, et al. Heart-Type Fatty Acid-Binding Protein (H-FABP) and its Role as a Biomarker in Heart Failure: What Do We Know So Far? *J Clin Med* 2020;**9**.

19. Zhuang L, Mao Y, Liu Z, Li C, Jin Q, Lu L, et al. FABP3 Deficiency Exacerbates Metabolic Derangement in Cardiac Hypertrophy and Heart Failure via PPARalpha Pathway. *Frontiers in cardiovascular medicine* 2021;**8**:722908.

20. Miao Y, Kang X. Evaluation of left ventricular function in patients with heart failure after myocardial infarction by real-time three-dimensional transesophageal echocardiography. *Am J Transl Res* 2021;**13**:10380-7.

21. Rodríguez-Calvo R, Granado-Casas M, Pérez-Montes de Oca A, Julian MT, Domingo M, Codina P, et al. Fatty Acid Binding Proteins 3 and 4 Predict Both All-Cause and Cardiovascular Mortality in Subjects with Chronic Heart Failure and Type 2 Diabetes Mellitus. *Antioxidants (Basel)* 2023;**12**.

22. Ahmad F, Karim A, Khan J, Qaisar R. Plasma Galectin-3 and H-FABP correlate with poor physical performance in patients with congestive heart failure. *Exp Biol Med (Maywood)* 2023;**248**:532-40.

23. Lichtenauer M, Jirak P, Wernly B, Paar V, Rohm I, Jung C, et al. A comparative analysis of novel cardiovascular biomarkers in patients with chronic heart failure. *Eur J Intern Med* 2017;**44**:31-8.

24. Courbebaisse M, Lanske B. Biology of Fibroblast Growth Factor 23: From Physiology to Pathology. *Cold Spring Harb Perspect Med* 2018;**8**.

25. Hao H, Li X, Li Q, Lin H, Chen Z, Xie J, et al. FGF23 promotes myocardial fibrosis in mice through activation of β-catenin. *Oncotarget* 2016;**7**:64649-64.

26. Pandhi P, Ter Maaten JM, Anker SD, Ng LL, Metra M, Samani NJ, et al. Pathophysiologic Processes and Novel Biomarkers Associated With Congestion in Heart Failure. *JACC Heart Fail* 2022;**10**:623-32.

27. Ceelen D, Voors AA, Tromp J, van Veldhuisen DJ, Dickstein K, de Boer RA, et al. Pathophysiological pathways related to high plasma growth differentiation factor 15 concentrations in patients with heart failure. *Eur J Heart Fail* 2022;**24**:308-20.

28. Roy C, Lejeune S, Slimani A, de Meester C, Ahn As SA, Rousseau MF, et al. Fibroblast growth factor 23: a biomarker of fibrosis and prognosis in heart failure with preserved ejection fraction. *ESC Heart Fail* 2020;**7**:2494-507.

29. Kanagala P, Arnold JR, Khan JN, Singh A, Gulsin GS, Eltayeb M, et al. Fibroblast-growth-factor-23 in heart failure with preserved ejection fraction: relation to exercise capacity and outcomes. *ESC Heart Fail* 2020.

30. Vergaro G, Del Franco A, Aimo A, Gentile F, Castiglione V, Saponaro F, et al. Intact fibroblast growth factor 23 in heart failure with reduced and mildly reduced ejection fraction. *BMC Cardiovasc Disord* 2023;**23**:433.

31. Yu L, He R, Wang D, Qi D. Activated Clec4n(hi) Neutrophils Aggravate Lung Injury in an Endothelial IGFBP7 Dependent Manner. *Am J Respir Cell Mol Biol* 2024.

32. Ko T, Nomura S, Yamada S, Fujita K, Fujita T, Satoh M, et al. Cardiac fibroblasts regulate the development of heart failure via Htra3-TGF-β-IGFBP7 axis. *Nat Commun* 2022;**13**:3275.

33. Paulus WJ, Zile MR. From Systemic Inflammation to Myocardial Fibrosis: The Heart Failure With Preserved Ejection Fraction Paradigm Revisited. *Circ Res* 2021;**128**:1451-67.

34. Gandhi PU, Gaggin HK, Sheftel AD, Belcher AM, Weiner RB, Baggish AL, et al. Prognostic usefulness of insulin-like growth factor-binding protein 7 in heart failure with reduced ejection fraction: a novel biomarker of myocardial diastolic function? *The American Journal of Cardiology* [Randomized Controlled Trial Research Support, Non-U.S. Gov't] 2014;**114**:1543-9.

35. Hage C, Bjerre M, Frystyk J, Gu HF, Brismar K, Donal E, et al. Comparison of Prognostic Usefulness of Serum Insulin-Like Growth Factor-Binding Protein 7 in Patients With Heart Failure and Preserved Versus Reduced Left Ventricular Ejection Fraction. *Am J Cardiol* 2018.

36. Katoh M, Nomura S, Yamada S, Ito M, Hayashi H, Katagiri M, et al. Vaccine Therapy for Heart Failure Targeting the Inflammatory Cytokine Igfbp7. *Circulation* 2024;**150**:374-89.

37. Ahmed A, Ahmed S, Arvidsson M, Bouzina H, Lundgren J, Radegran G. Elevated plasma sRAGE and IGFBP7 in heart failure decrease after heart transplantation in association with haemodynamics. *ESC Heart Fail* 2020;**7**:2340-53.

38. Kalayci A, Peacock WF, Nagurney JT, Hollander JE, Levy PD, Singer AJ, et al. Echocardiographic assessment of insulin-like growth factor binding protein-7 and early identification of acute heart failure. *ESC Heart Fail* 2020;**7**:1664-75.

39. Shah AM, Myhre PL, Arthur V, Dorbala P, Rasheed H, Buckley LF, et al. Large scale plasma proteomics identifies novel proteins and protein networks associated with heart failure development. *Nat Commun* 2024;**15**:528.

40. Gandhi PU, Chow SL, Rector TS, Krum H, Gaggin HK, McMurray JJ, et al. Prognostic Value of Insulin-Like Growth Factor-Binding Protein 7 in Patients with Heart Failure and Preserved Ejection Fraction. *J Cardiac Fail* 2017;**23**:20-8.

41. Motiwala SR, Szymonifka J, Belcher A, Weiner RB, Baggish AL, Gaggin HK, et al. Measurement of novel biomarkers to predict chronic heart failure outcomes and left ventricular remodeling. *J Cardiovasc Transl Res* [Research Support, Non-U.S. Gov't] 2014;**7**:250-61.

42. Bracun V, van Essen B, Voors AA, van Veldhuisen DJ, Dickstein K, Zannad F, et al. Insulin-like growth factor binding protein 7 (IGFBP7), a link between heart failure and senescence. *ESC Heart Fail* 2022;**9**:4167-76.

43. Ferreira JP, Packer M, Sattar N, Butler J, Gonzalez Maldonado S, Panova-Noeva M, et al. Insulin-like growth factor binding protein-7 concentrations in chronic heart failure: Results from the EMPEROR programme. *Eur J Heart Fail* 2024;**26**:806-16.

44. El-Armouche A, Pohlmann L, Schlossarek S, Starbatty J, Yeh YH, Nattel S, et al. Decreased phosphorylation levels of cardiac myosin-binding protein-C in human and experimental heart failure. *J Mol Cell Cardiol* 2007;**43**:223-9.

45. Rosas PC, Warren CM, Creed HA, Trzeciakowski JP, Solaro RJ, Tong CW. Cardiac Myosin Binding Protein-C Phosphorylation Mitigates Age-Related Cardiac Dysfunction: Hope for Better Aging? *JACC Basic Transl Sci* 2019;**4**:817-30.

46. Tamargo M, Martínez-Legazpi P, Espinosa M, Lyon A, Méndez I, Gutiérrez-Ibañes E, et al. Increased Chamber Resting Tone Is a Key Determinant of Left Ventricular Diastolic Dysfunction. *Circ Heart Fail* 2023;**16**:e010673.

47. Mohamed IA, Krishnamoorthy NT, Nasrallah GK, Da'as SI. The Role of Cardiac Myosin Binding Protein C3 in Hypertrophic Cardiomyopathy-Progress and Novel Therapeutic Opportunities. *J Cell Physiol* 2017;**232**:1650-9.

48. Heling L, Geeves MA, Kad NM. MyBP-C: one protein to govern them all. *Journal of muscle research and cell motility* 2020;**41**:91-101.

49. Kozhuharov N, Wussler D, Kaier T, Strebel I, Shrestha S, Flores D, et al. Cardiac myosin-binding protein C in the diagnosis and risk stratification of acute heart failure. *Eur J Heart Fail* 2021;**23**:716-25.

50. Chetran A, Badescu MC, Serban IL, Duca ST, Afrasanie I, Cepoi MR, et al. Insights into the Novel Cardiac Biomarker in Acute Heart Failure: Mybp-C. *Life (Basel)* 2024;**14**.

51. Kaier TE, Twerenbold R, Puelacher C, Marjot J, Imambaccus N, Boeddinghaus J, et al. Direct Comparison of Cardiac Myosin-Binding Protein C With Cardiac Troponins for the Early Diagnosis of Acute Myocardial Infarction. *Circulation* 2017;**136**:1495-508.
