# Supplementary material for: Novel biomarkers distinguish heart failure with preserved vs reduced ejection fraction
Source: ESC Heart Fail. 2026 Jan 8;13(3):xvaf011. doi: 10.1093/eschf/xvaf011 (PMC13228998; doi:10.1093/eschf/xvaf011)
Supplement: xvaf011_Supplementary_Data [file xvaf011_supplementary_data.zip › Supplemental Table 1 Biomarker assays 22MAY2025.docx]

**Supplemental Table 1**

*Biomarker assays*

| NT-proBNP | Analysed by the fully automated quantitative Elecsys® proBNP II STAT assay using a cobas**®** e 601 analyzer (Roche Diagnostics, Bromma, Sweden) |
| --- | --- |
| Copeptin, MR-proADM and MR-proANP | Analyzed by a commercially available automated immunofluorescent assay on a Kryptor Compact Plus (BRAHMS, Henningsdorf/Berlin, Germany |
| Leptin and adiponectin | Analyzed by a radio immunoassay (RIA) Merk Millipore® (HL-81 K and HADP-61 K) |
| Serum IGF-I and IGFBP-1 | Analyzed by in-house radioimmunoassays at the Department of Endocrinology at Karolinska University Hospital  IGF-I values were expressed as age adjusted standard deviation (SD) scores calculated from the regression of the IGF-I concentrations of healthy adult subjects (SD score=((10lnIGF-I-observed+ 0.00693*age)-2.581)/0.120) |
| Insulin | Analyzed with ELISA K6219 (Dako cytomation, Stockholm, Sweden)  Insulin resistance was assessed according to homeostatic model assessment (HOMA-IR) calculated as ([glucose*insulin]/22.5; with glucose in mmol/L and insulin in mU/L) |
| sS2 | Analyzed by using the Critical Diagnostics Presage® ST2 Assay kit that quantitatively measures sST2 by enzyme-linked immunosorbant assay (ELISA) in a microtiter plate format |
| Galectin 3 | Analyzed by KE00126, human Galectin 3 ELISA kit, Nordic Biosite |

MRproANP=MR-pro-atrial natriuretic peptide; MR-proADM=MR-pro-adrenomedullin; NT-proBNP=N-terminal pro-brain natriuretic peptide; sST2=Soluble suppression of tumorigenecity
