# Supplementary figures and images for: Novel biomarkers distinguish heart failure with preserved vs reduced ejection fraction
Source: ESC Heart Fail. 2026 Jan 8;13(3):xvaf011. doi: 10.1093/eschf/xvaf011 (PMC13228998; doi:10.1093/eschf/xvaf011)

Supplemental Figure 2.

Correlations between NYHA class biomarkers in HFpEF and HFrEF


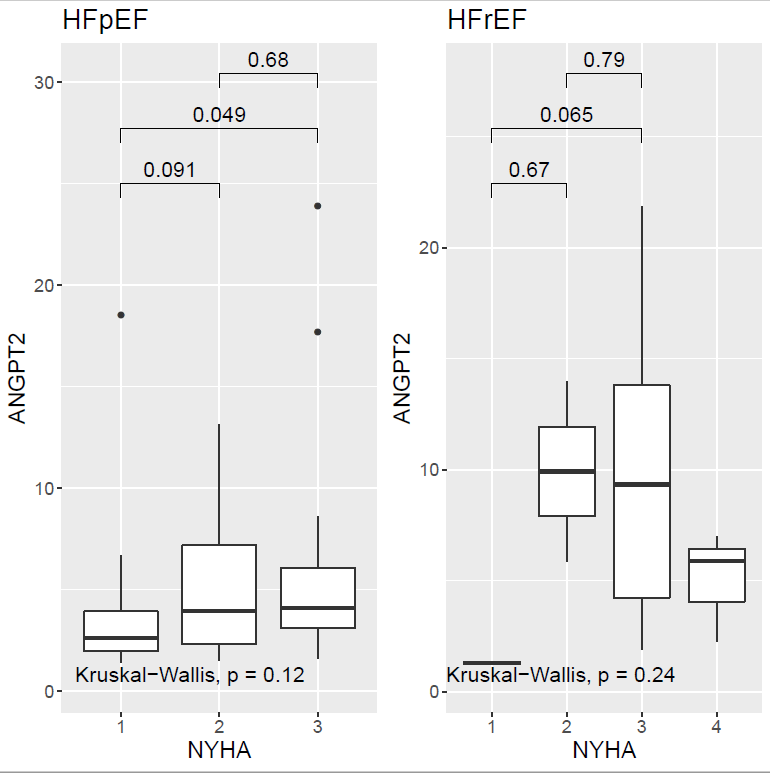

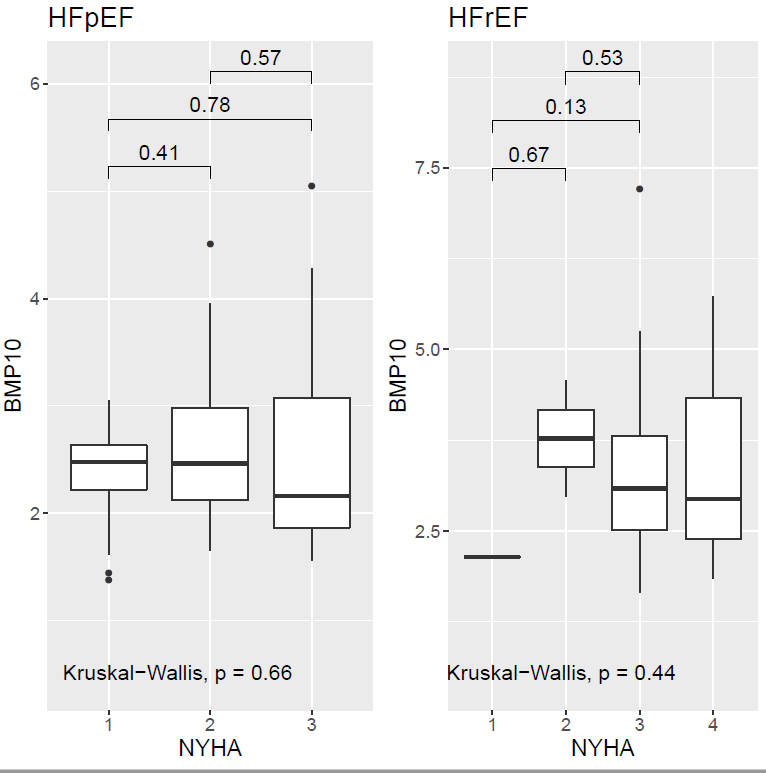


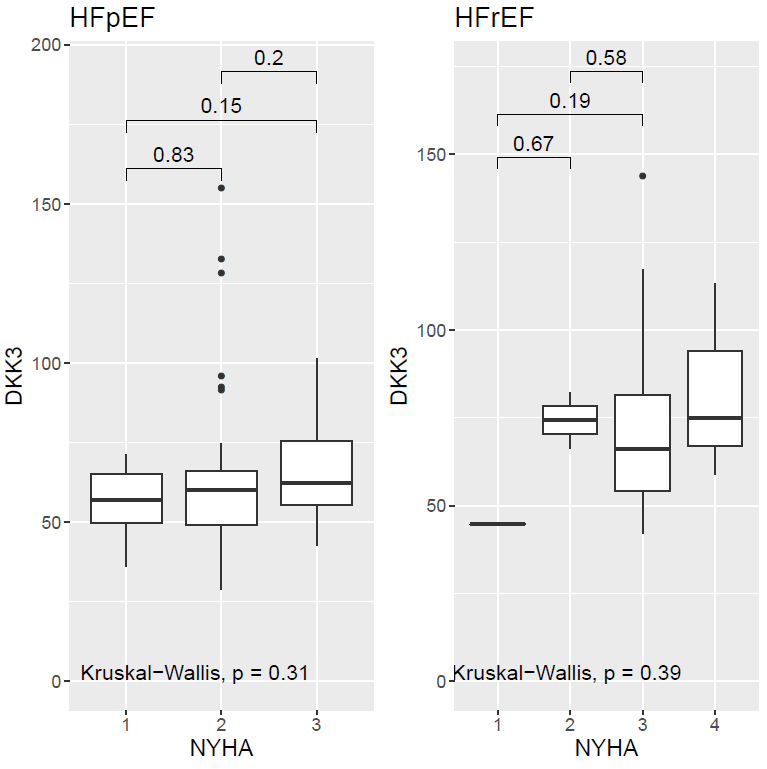

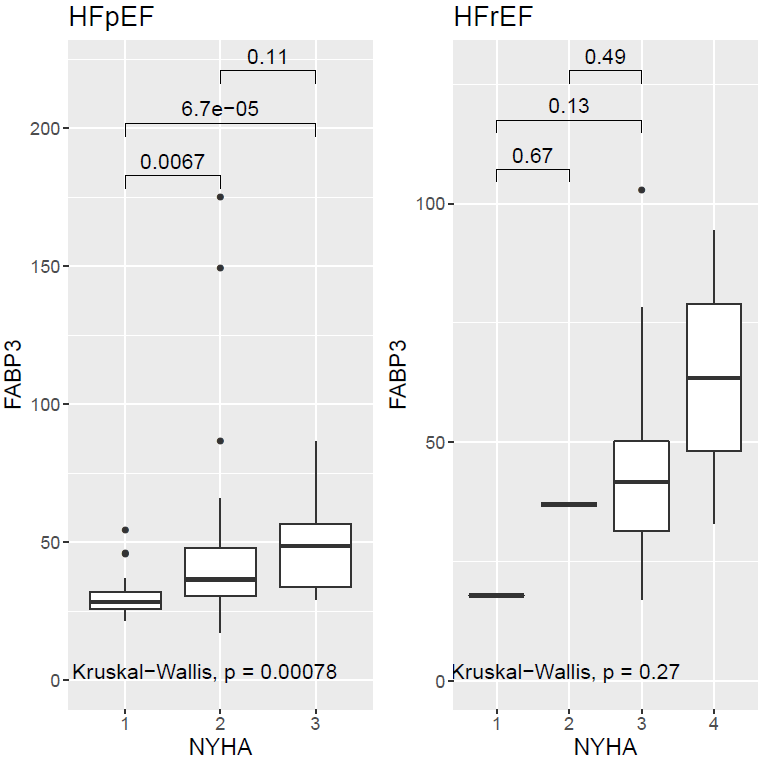


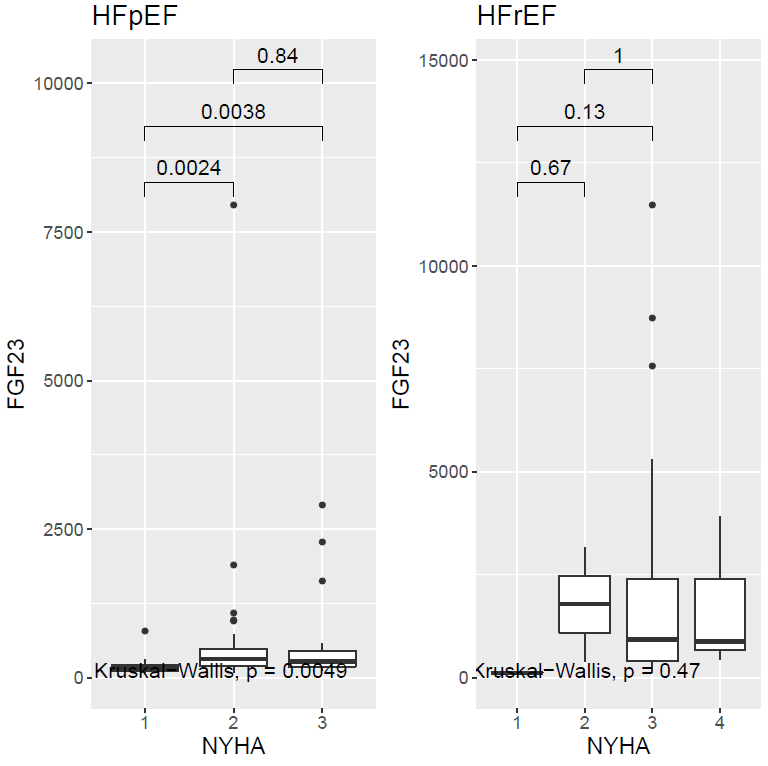

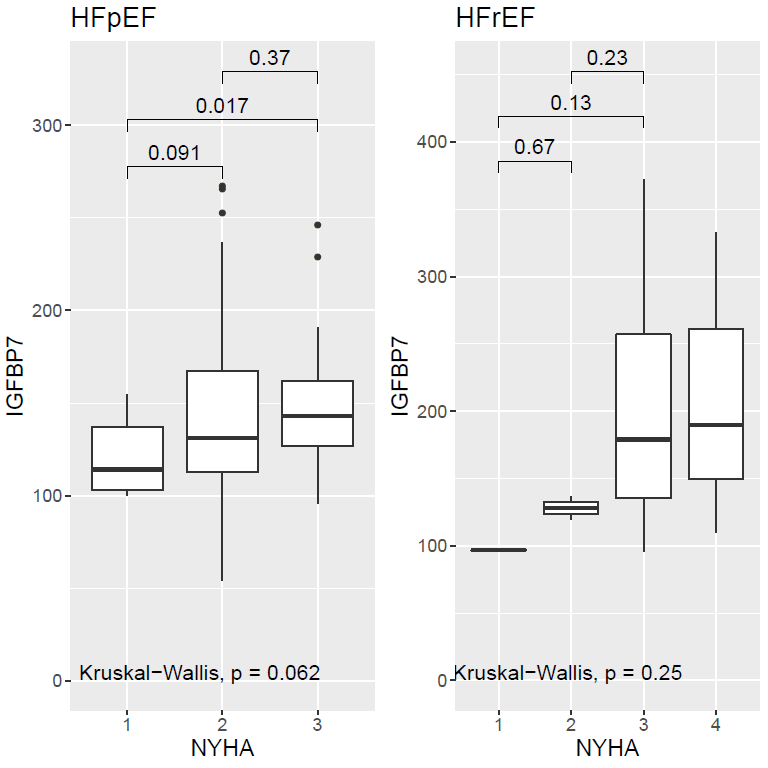


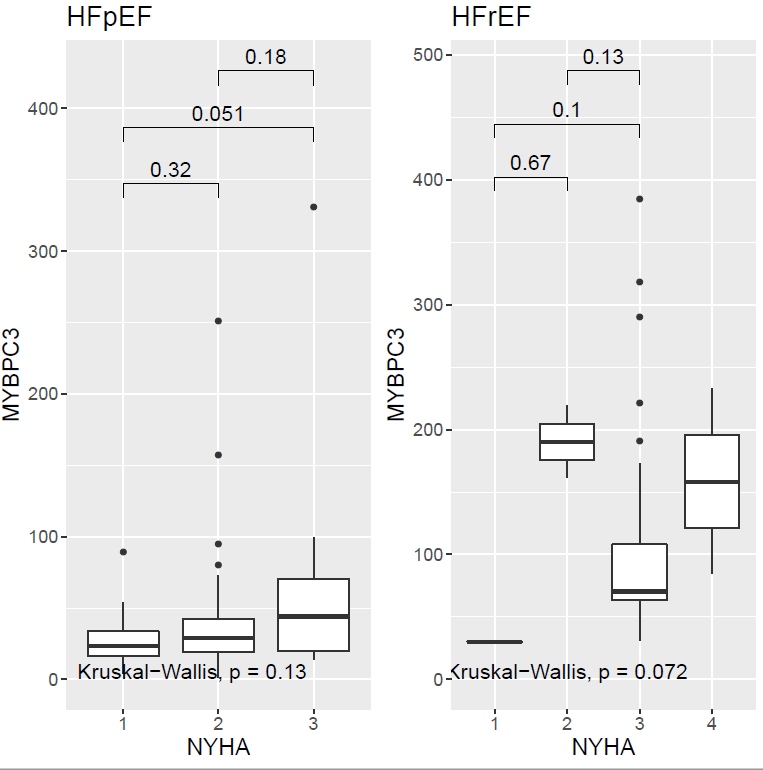

Supplement: xvaf011_Supplementary_Data [file xvaf011_supplementary_data.zip › Supplemental Figure 2 22MAY2025.docx]
